# Genetic and chromatin regulation of *Pvt1* monoallelic expression

**DOI:** 10.1016/j.celrep.2025.116554

**Published:** 2025-11-11

**Authors:** Christy Luong, Mason Chen, Julia A. Belk, Katerina Kraft, Anne-Valerie Gendrel, Edith Heard, Joanna Wysocka, Howard Y. Chang

**Affiliations:** 1 Departments of Dermatology and Genetics, Stanford University School of Medicine, Stanford, CA 94305, USA; 2 Department of Chemical and Systems Biology, Stanford University School of Medicine, Stanford, CA 94305, USA; 3 Gulbenkian Institute for Molecular Medicine, 1649-028 Lisbon, Portugal; 4 European Molecular Biology Laboratory, Director’s Research, 69117 Heidelberg, Germany; 5 Collège de France, 75005 Paris, France; 6 Department of Developmental Biology, Stanford University School of Medicine, Stanford, CA 94305, USA; 7 Institute for Stem Cell Biology and Regenerative Medicine, Stanford University School of Medicine, Stanford, CA 94305, USA; 8 Howard Hughes Medical Institute, Stanford University, Stanford, CA 94305, USA; 9 Department of Pathology, Stanford University School of Medicine, Stanford, CA 94305, USA; 10 Present address: Amgen Research, South San Francisco, CA 94080, USA; 11 Lead contact

## Abstract

While most genes are equivalently expressed on both alleles, genes with random monoallelic expression (RME) stably maintain expression from only one allele, but the mechanisms and consequences of RME remain unclear. We performed allele-specific RNA sequencing (RNA-seq) on ∼100 F_1_ hybrid neural progenitor cell (NPC) clonal lines to reveal the extent of autosomal RME (aRME). Of the 287 aRME genes, *Pvt1*, an oncogenic long non-coding RNA, is an aRME with a genetic bias. In the absence of genetic differences, *Pvt1* undergoes balanced aRME. *Pvt1* monoallelic expression is maintained by allele-specific active and repressive histone modifications, opposed to DNA methylation. Additionally, we provide a two-step mechanism for the initiation of aRME and demonstrate that *Pvt1* monoallelic expression results in a growth phenotype due to the interplay with *Myc*. These findings provide insight into how genetic differences can skew a stochastic process, resulting in monoallelic expression with a phenotypic consequence in early development.

## In brief

Luong et al. reveal how genetic variation affects autosomal random monoallelic expression (aRME) of *Pvt1* via differential TFAP2a binding. The stochastic allelic choice for aRME is propagated through cell differentiation through chromatin-based mechanisms, and *Pvt1* monoallelic expression regulates growth via in *cis* interactions with *Myc*, offering insights into aRME function.

## Graphical Abstract

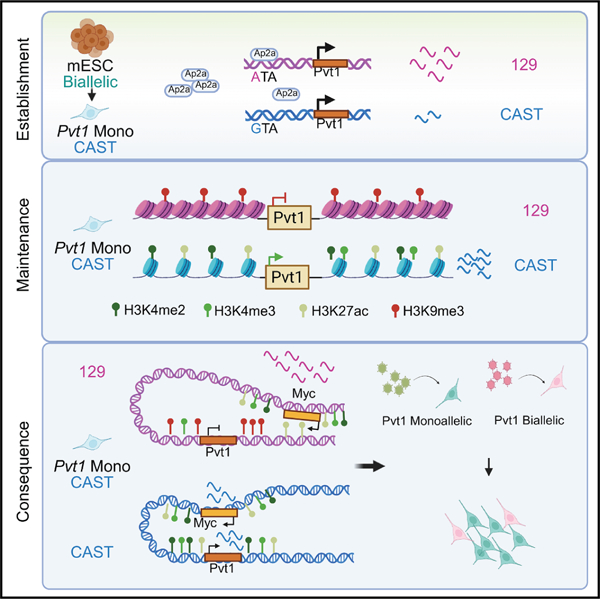

## INTRODUCTION

In diploid organisms, most genes are expressed from both maternal and paternal alleles. However, a subset of genes shows monoallelic expression (ME), where one allele is actively transcribing and the other allele is silenced. ME is an epigenetic phenomenon where the DNA sequence is not altered, but monoallelic expression is stably maintained over cell division and differentiation. Genes with ME can be deterministic, as in imprinted genes with parental epigenetic marks that control gene expression during development,^1–3^ or random monoallelic expression (RME), such as X chromosome inactivation (XCI) in females.^4,5^ Other well-studied groups of RME genes include olfactory receptors^6,7^ and immunoglobulin receptors,^8–10^ where RME leads to receptor diversity,^11^ and protocadherin genes that shape neuronal identities.^12^ Unbiased studies show that 0.5%–10% of genes have autosomal RME (aRME) in certain cell types,^6,11,13^ where the expression pattern is stably maintained with somatic cell division and is independent of parental origin.

aRME genes increase upon mouse embryonic stem cell (mESC) differentiation,^14^ suggesting that aRME is established during differentiation. Many aRME genes have bi-allelically accessible chromatin in mESCs but become monoallelically accessible as they differentiate to neural progenitor cells (NPCs) with H3K4me3 on active alleles and H3K9me3 on inactive alleles.^15,16^ However, there remain gaps in our knowledge about the mechanisms of aRME and whether aRME regulates precise gene expression during early development. This study aims to catalog aRME genes and elucidate the mechanisms by which aRME is established and maintained over cell division, as well as the functional consequences of having an aRME gene.

Over 100 NPC clonal lines were analyzed to catalog aRME genes, highlighting *Pvt1* for its notable properties as a highly expressed oncogenic long non-coding RNA (lncRNA) in induced pluripotent stem cells (iPSCs).^17^ *Pvt1* is bi-allelically expressed in mESCs but becomes ME in NPCs.^15^ Although *Pvt1* has not been well studied in the context of development, during the oocyte-to-embryo transition, *Pvt1* is shown to be significantly upregulated.^18^ *PVT1* is highly expressed in human iPSCs, and the expression of *PVT1* significantly decreases with neuronal differentiation,^17^ indicating a role in stemness and cell identity. In cancer, *PVT1* is well studied as a cancer mutation hotspot and oncogenic locus that drives proliferation during tumorigenesis with its neighboring gene, *MYC*,^19,20^ known to be involved in cell proliferation, growth, and cell competition.^21–23^ The tight regulation of *Myc* is critical for ensuring neural cell fate transition.^21^ Additionally, the *PVT1* promoter, independent of the *PVT1* lncRNA, acts as a tumor suppressor by decreasing the expression of *MYC* via enhancer competition with intragenic enhancers within *PVT1*.^24–26^

In this study, we performed genome-wide RNA sequencing (RNA-seq) and epigenomic profiling to identify and quantify a robust list of aRME genes in over 100 NPC clonal lines, examining their allelic expression and chromatin signatures. By leveraging this large dataset, *Pvt1* demonstrates genetically driven skewed aRME, enabling investigation of the initiation and allelic choice mechanism. Furthermore, *Pvt1* ME correlates with a growth phenotype that is likely a conseqeunce of enhancer-promoter competition with *Myc* and increased *Pvt1* expression.

## RESULTS

### Allele-specific RNA-seq of NPC clonal lines

**Figure 1.**
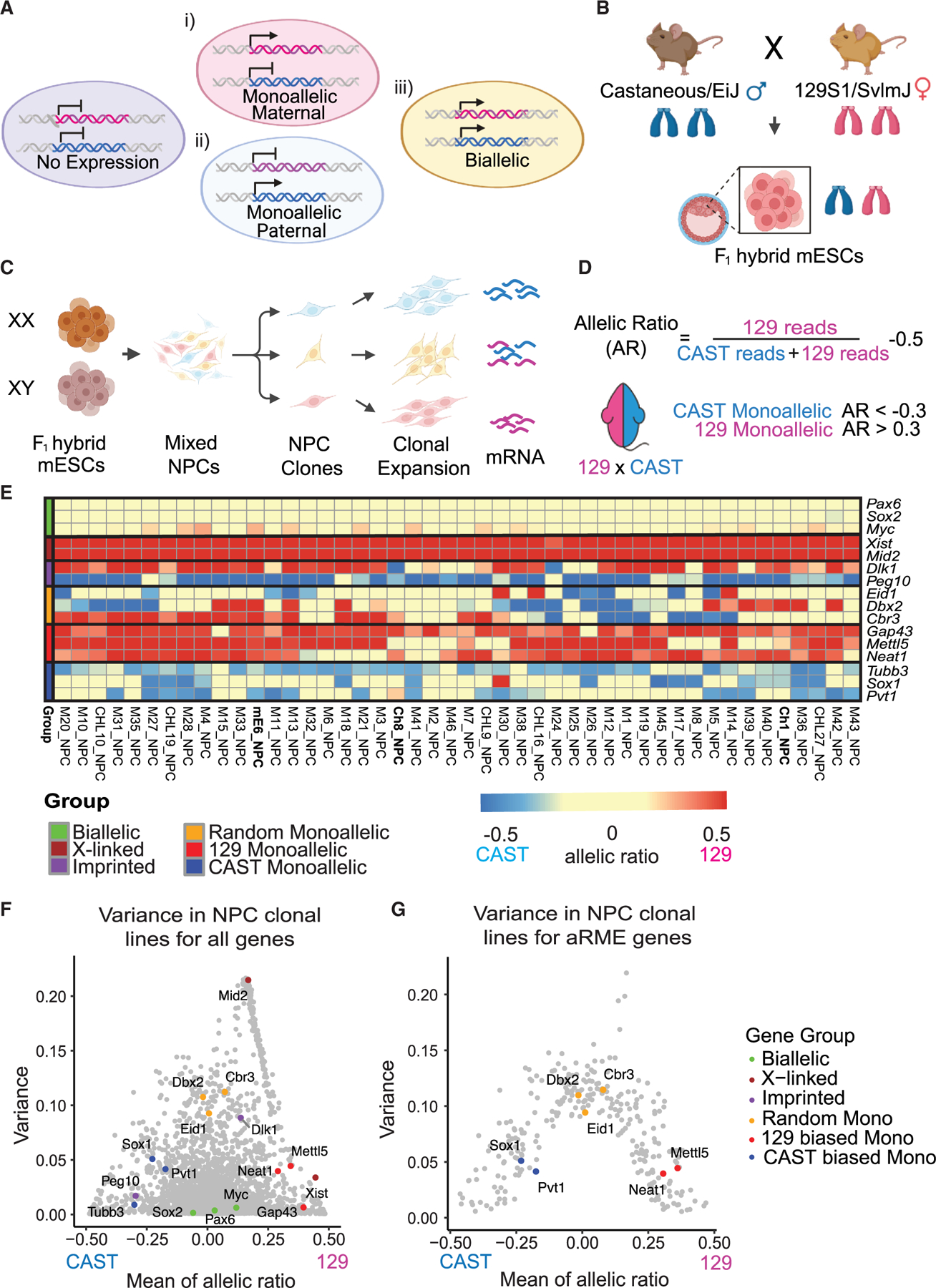
Allele-specific RNA-seq on F1 hybrid NPC clonal lines (A) Schematic of possibilities in gene expression in a single cell: no gene expression (purple), monoallelic expression from the maternal allele (pink), monoallelic expression from the paternal allele (blue), and bi-allelic expression from both alleles (yellow). (B) Schematic of model system. F_1_ hybrid mouse ESCs (mESCs) derived from a cross between a male castaneous/EiJ mouse and a female 129S1/SvlmJ mouse. (C) Schematic of the differentiation protocol from mESCs to NPCs. (D) Calculations for the allelic ratio (AR) of a single gene. (E) Heatmap of the ARs of different genes across male NPC clonal lines. The heatmap is colored from CAST biased (blue) to bi-allelic (yellow) to 129 biased (red). On the *x* axis is the name of each male clonal line (*n* = 46). (F) A plot of variance against the mean of AR for all genes expressed across all the female and male NPC clonal lines (*n* = 120). (G) A plot of variance against the mean of AR for aRME genes across all female and male NPC clonal lines.

Most genes are bi-allelically expressed or not expressed at all in certain cell types. However, a few genes are stably monoallelically expressed (ME) and can be found in three states in single cells: ME from the maternal allele, ME from the paternal allele, or bi-allelic expression from both alleles (Figure 1A). Monoallelically expressed genes maintain these states through cell division, so clonal lines from a single cell will consistently display the same allele-specific expression pattern. In comparison, normal genes will only have stable bi-allelic expression in clonal lines. This variation in gene expression states of genes with ME generates cellular heterogeneity during early development.

To assess allele-specific expression, clonal NPC lines were differentiated from F_1_ hybrid mESCs, derived from a 129S1(129) x CAST/EiJ (CAST) cross^6,15,16^ (Figure 1B). With approximately 24 million single-nucleotide polymorphisms (SNPs) (∼1 SNP every 100 bp) between these strains, gene expression and chromatin state from the maternal and paternal alleles can be tracked at high resolution.^6,15^ Briefly, male and female F_1_ hybrid mESCs were differentiated into NPCs, which were subcloned to make clonal lines for studying stable ME maintained with cell division (Figure 1C). This approach can distinguish ME from transient transcriptional bursting effects since all cells in an monoallelically expressing clonal line share the same allelic choice, while bi-allelic genes remain bi-allelic in clonal lines.

To quantify ME in NPCs, we performed allele-specific bulk RNA-seq on 46 male and 74 female NPC clonal lines (Figures 1C and S1A). Bulk RNA-seq averages out transcriptional bursting seen at the single-cell level. Sequencing reads were mapped to an N-masked reference genome and split using informative SNPs (Figure S1B), with non-informative reads excluded. The allelic ratio (AR) of the gene expression is calculated as the aligned 129 reads divided by the sum of CAST and 129 reads, minus 0.5 (Figure 1D). ARs of −0.5, 0, and +0.5 indicate exclusive CAST, equal, and exclusive 129 allele expression, respectively.

### Overview of monoallelic expressing genes

To identify aRME genes, we filtered for genes with an average transcript per million (TPM) count greater than 5 in NPCs, yielding 10,318 genes (Figure S1C). Each gene in each NPC clonal line was classified as monoallelic or bi-allelic using AR ≥ 0.3 and AR ≤ −0.3, based on X-linked gene benchmarks.^15^ Genes were defined as monoallelically expressed if they were monoallelic, as opposed to bi-allelic, in at least 40% of the NPC clonal lines (ME cutoff of 0.4; 954 genes) (Figure S1C). As AR and ME cutoffs can differ by analysis, a list with varying AR and ME thresholds is provided (Table S1). After excluding all X- and Y-linked and imprinted genes,^27^ we focus on autosomal ME (aME) genes. To consider a gene aRME, it must have CAST-monoallelic expression (AR ≤ −0.3) in at least one clonal line and 129-monoallelic expression (AR ≥ 0.3), resulting in 287 aRME gene candidates (2.7% of expressed genes) (Figure S1C; Table S2), fewer than what has previously been reported.^6,14^ A broader list of 638 aME genes (6.1%) includes aRME, as well as aME genes that do not meet the previous requirement to be called random ME (RME). These genes may include unclassified imprinted genes and RME genes that show strong genetic biases in our F_1_ hybrid model system.

Known bi-allelic, X-linked, and imprinted genes validated the dataset (Figures 1E and S1D). In male NPCs, *Xist* and *Mid2* are only expressed from the maternal X (Figure 1E); in female NPCs, *Xist* can be randomly monoallelic with a 129 genetic bias, and *Mid2* is expressed on the active X allele, with a small fraction showing loss of the paternal X (Figure S1D). Interestingly, *Dlk1* is normally a paternally expressed gene^28^ but is maternally expressed in the neural niche^29^ and in our NPC clonal lines. Some clones also show a loss of imprinting for some genes, which is known to occur in mESCs cultured in 2i+LIF (2i: MEK and GSK3 inhibitors; LIF: leukemia inhibitory factor) conditions^30^ (Figure 1E).

Of note, aRME genes showed a gradation of allelic bias in our hybrid line. True aRME genes (*Eid1*, *Dbx2*, and *Cbr3*) have robust monoallelic or bi-allelic expression across clonal lines, while others display an allele-specific bias (129: *Gap43*, *Mettl5*, and *Neat*; *CAST*: *Tubb3*, *Sox1*, and *Pvt1*; Figures 1E and S1D). To visualize the variation of all expressed genes and their gene group classification, we plotted the mean of AR for each gene against the variance across all male and female NPC clonal lines (Figure 1F). Most normal bi-allelic genes, such as *Sox2* and *Pax6*, will have a mean AR of zero and no variance. aRME genes have a mean AR of approximately zero with high variance, and ME genes with genetic biases have a non-zero AR mean and lower variance (Figure 1F). There is diversity in the extent of RME for each gene, with some genes showing genetic biases (Figure 1G). Filtering for ME from both CAST and 129 in separate clonal lines excludes genes with a bias toward only one allele, such as *Tubb3* and *Gap43*, which are considered aME, rather than aRME, genes (Figures 1F and 1G).

### Epigenomic profile of monoallelic expressing genes

**Figure 2.**
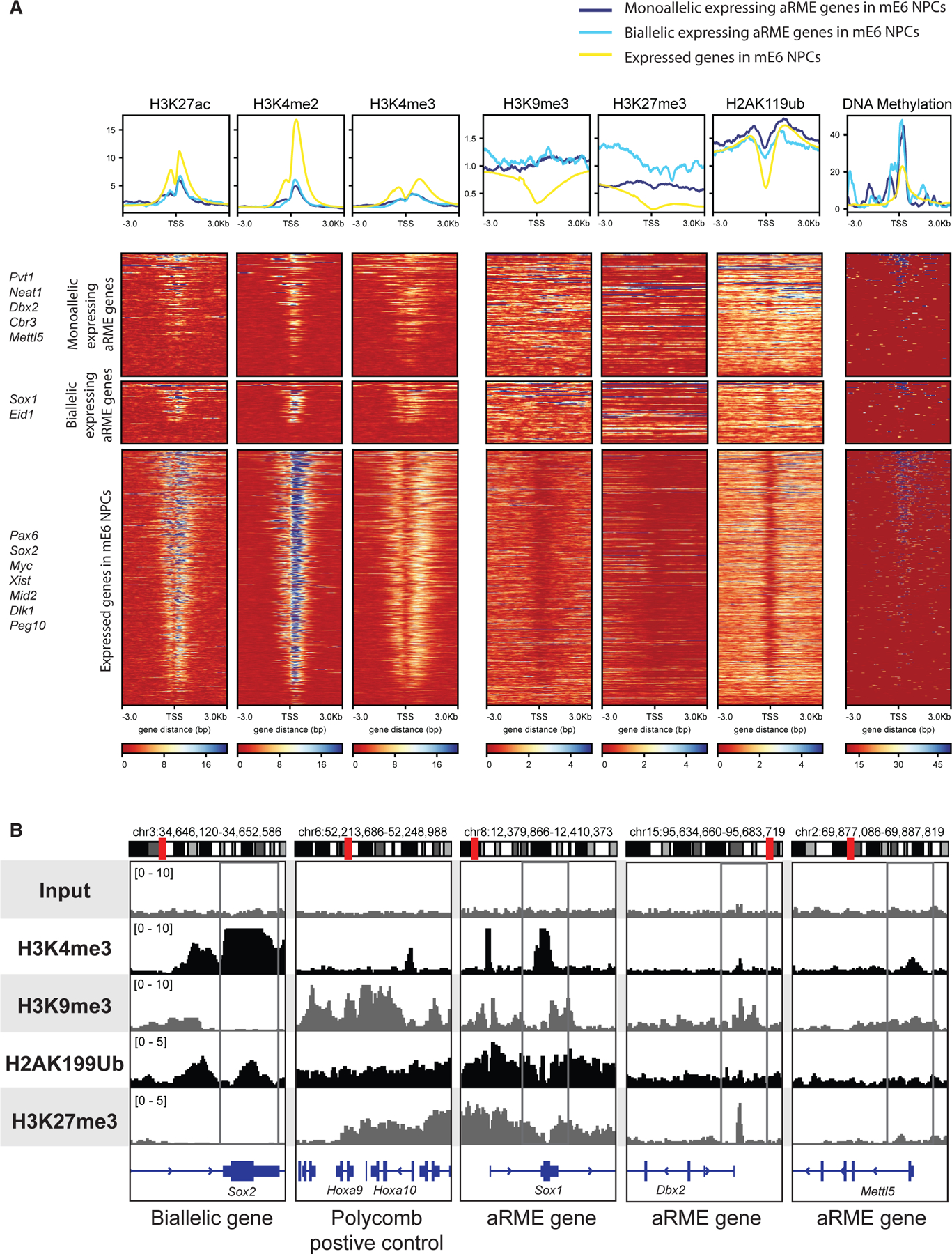
Epigenomic profile of monoallelic expressing genes (A) Combined heatmap of chromatin immunoprecipitations (ChIPs) of epigenetic markers and bisulfite sequencing in the mE6 NPC clonal line. At the top is a summary plot of ChIP signals from the three groups of genes: monoallelic expressing aRME genes, bi-allelic expressing aRME genes, and all non-aRME genes expressed. On the left of the heatmap are some genes that can be found in each group. At the bottom is the scale in reads per genomic content (RPGC) for the ChIPs and counts per million (CPM) for DNA methylation. (B) H3K4me3, H3K9me3, H2AK119Ub, and H3K27me3 ChIP-seq in mE6 NPCs. *Sox2* is a normal bi-allelic expressing gene, a negative control for repressive marks. *Hoxa9/10* are positive controls for repressive marks. *Sox1* is an aRME gene with bi-allelic expression in mE6 NPCs. *Dbx2* and *Mettl5* are aRME genes with monoallelic expression in mE6 NPCs. The data range is in RPGC.

After narrowing down aRME genes, we next examined which epigenetic marks associate with their ME maintenance during cell division. To this end, we performed allele-specific chromatin immunoprecipitation sequencing (ChIP-seq) for active and repressive histone modifications in three different F_1_-23 NPC clonal lines: mE6 NPCs, Ch1 NPCs, and Ch8 NPCs (Figure S1A). To broadly compare the allelic expression status of aRME and their epigenetic marks, we examined the active marks H3K27ac, H3K4me2, and H3K4me3, as well as the repressive marks H3K9me3, H2AK199ub, and H3K27me3. ChIP signals for active marks were generally lower in monoallelic expressing aRME genes compared to normal bi-allelic genes. Notably, monoallelic and bi-allelic expressing aRME genes have the same active mark levels despite copy-number differences, hinting that active marks are not simply additive, and there is a separate regulatory control for aRME genes compared to normally expressed genes, potentially a compensatory mechanism (Figures 2A, S2A, and S2B).

Inversely, repressive histone marks and DNA methylation were higher overall for aRME genes, especially near the transcription start site (TSS), with H3K27me3 showing the most pronounced difference between monoallelic and bi-allelic expressing aRME genes (Figures 2A and S2A). Bi-allelic aRME genes had more H3K27me3 signals than monoallelic aRME genes, except in the Ch8 clonal line, possibly due to differences in ME status (Figures 2A, S2A, and S2B). A lack of co-clustering between DNA methylation and histone modifications for aRME genes indicates minimal overlap, suggesting that aRME genes may be maintained by different epigenetic mechanisms.

When we look at specific gene loci such as *Sox1*, an aRME gene, with strong H3K9me3, H2AK199Ub, and H3K27me3 signals, this suggests that monoallelic expression is controlled by both H3K9me3 and Polycomb complexes (Figure 2B). In contrast, *Sox2*, a normal bi-allelic expressing gene, lacks both H3K9me3 and H3K27me3 marks along the gene body but is marked by H2AK199ub. However, not all aRME genes are marked by repressive histone modifications. For example, *Mettl5* lacks these marks but is enriched for DNA methylation downstream of the TSS (Figure S3F). Overall, aRME gene maintenance involves subtle and varying epigenetic mechanisms depending on the gene.

### *Pvt1* has a CAST-biased monoallelic expression

**Figure 3.**
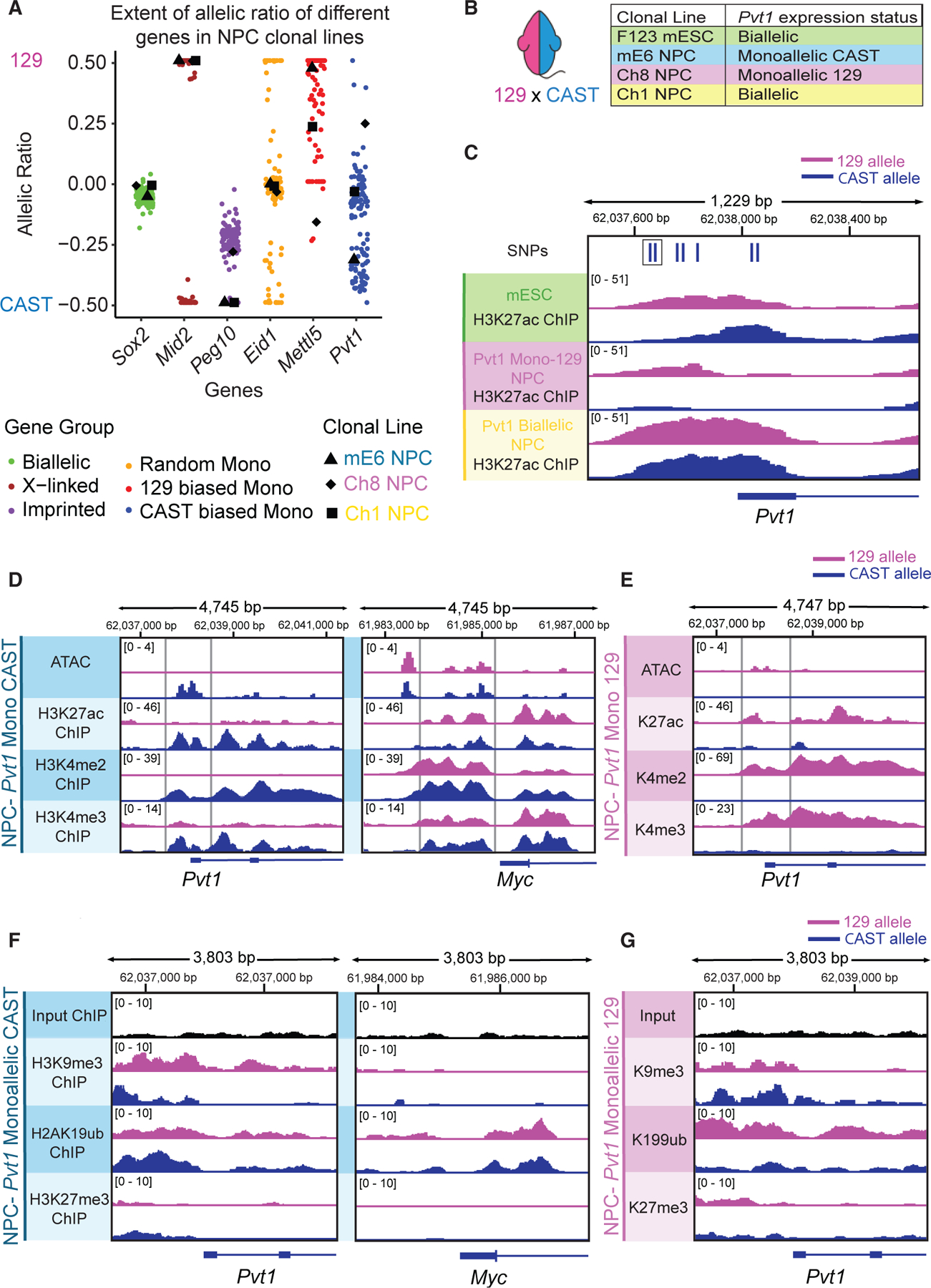
Chromatin landscape of Pvt1 monoallelic expressing clonal lines (A) Allelic ratio (AR) of *Pvt1* in F_1_-23 hybrid NPC clonal lines compared to other genes. Each point represents a clonal line (*n* = 120). NPC clonal lines used for other experiments are highlighted with different shapes. (B) Summary of F_1_ hybrid clonal lines used in this paper and their respective *Pvt1* allelic expression status based on an AR cutoff of 0.2. (C) Allele-specific H2K27ac ChIP-seq with 3 different F_1_-23 clonal lines. Top row shows SNP differences between the 129 allele and the CAST allele in relation to the H3K27ac ChIP-seq signals across three samples. *Tfap2a* SNP is highlighted in the black box, the second of the two SNPs. (D) Allele-specific ATAC-seq and ChIP-seq from mE6 NPCs, clonal line with *Pvt1* CAST monoallelic expression (mE6 NPC, blue). (E) Allele-specific ATAC-seq and ChIP-seq from Ch8 NPCs, clonal line with *Pvt1* 129 monoallelic expression (Ch8 NPC, pink). (F) ChIP input tracks (black) with allele-specific ChIP-seq tracks from mE6 NPCs. (G) ChIP input track (black) with allele-specific ChIP-seq tracks from Ch8 NPCs.

RNA-seq analysis identified *Pvt1*, a well-studied oncogenic lncRNA located in an oncogenic locus,^19–21,23^ as exhibiting a pronounced monoallelic expression toward the CAST background in NPC clonal lines compared to other aRME genes (Figure 3A). 56 NPC clones monoallelically express the CAST allele, while only 4 clones monoallelically express the 129 allele, a 14-fold bias. When examining the extent of ME for a few selected genes, it becomes evident that each gene exhibits a distinct distribution, likely driven by the inherent properties of the gene and the influence of genetic differences. This highlights the importance of adjusting the AR cutoff based on the gene of interest (Figure S3A). In our case, we focused on *Pvt1* and chose ARs of −0.2 and 0.2 for downstream analysis, as this threshold effectively separates the clonal lines into three groups: monoallelic CAST, monoallelic 129, and bi-allelic.

### Chromatin landscape of *Pvt1* locus

Epigenomic analysis reveals that chromatin accessibility, measured by assay of transposase accessible chromatin by sequencing (ATAC-seq),^15^ and occupancy of the enhancer-associated mark H3K27ac of the *Pvt1* locus are allelically matched to expression status in each clonal line (Figures 3B–3E; Table S3). The silenced alleles lose accessibility and transcription upon differentiation from mESCs to NPCs (Figure 3C). In comparison, *Myc*, 50 kb upstream, remains bi-allelically expressed and accessible on both alleles (Figure 3D).

To further investigate the epigenetic landscape at the *Pvt1-Myc* locus, we performed allele-specific ChIP of histone modifications and RNA polymerase II (RNA Pol II) subunit RPB1, as well as bisulfite sequencing, on an NPC clonal line with *Pvt1* ME on the CAST allele (Figures 3D and 3E) or the 129 allele (Figures 3E and 3G) and a line with *Pvt1* bi-allelic expression (Figure 3E). Clonal lines with *Pvt1* ME show that the *Pvt1* promoter and gene body of the active allele are marked with H3K4me2 and H3K4me3 (Figures 3D and 3E), whereas the inactive allele is marked by H3K9me3 along the gene body (Figures 3F and 3G). The H3K9me3 signal is lower in the *Pvt1* bi-allelically expressing clonal line (Figure S3E), in comparison. Interestingly, both alleles for all three NPC clonal lines have an H2AK199ub signal upstream and downstream of the TSS of *Pvt1*. In mESCs, the H3K4me2/3 signal is on both alleles, and the H3K27me3 signal is present upstream/downstream of the TSS but is absent on the gene body in NPCs (Figure S3D), indicating a bivalent promoter^31,32^ that resolves upon differentiation. In contrast, the *Myc* promoter retains active marks and lacks repressive marks on both alleles (Figures 3D and 3F).

Bisulfite sequencing showed no DNA methylation at the *Pvt1-Myc* locus, unlike for ME genes, such as Mettl5 and *Dlk1* (Figure S3F). The inactive *Pvt1* allele has a higher occupancy of RNA Pol II at the promoter, indicating stalled RNA Pol II, while the active allele exhibits a lower occupancy of Pol II at the promoter region, suggestive of pause release, which is consistent with its allelic transcriptional output (Figure S3C). In a *Pvt1* biallelic NPC clonal line, RNA Pol II is present on both alleles, with a slightly higher signal on the 129 allele (Figure S3C). Overall, the data suggest that H3K4me2/3, H3K27ac, H3K9me3, and H2AK199ub are associated with the maintenance of *Pvt1* ME during cell division in NPCs.

### Restoration of *Pvt1* random monoallelic expression in the absence of genetic differences

**Figure 4.**
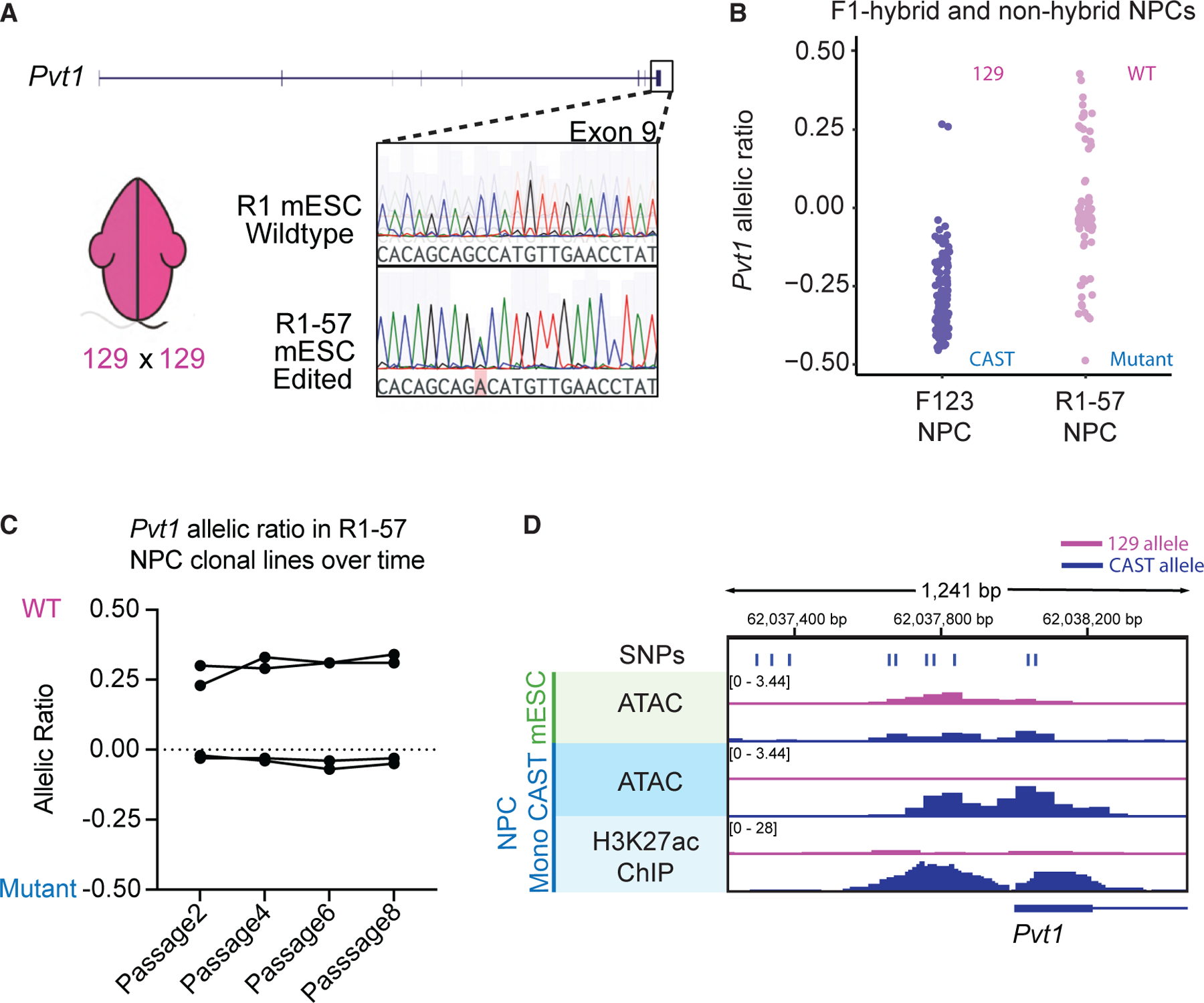
Pvt1 has balanced aRME in the absence of genetic differences (A) Model schematic of an R1 mESC with 129 × 129 background with a gene edit to contain a single SNP on *Pvt1* exon 9 (R1-57 mESCs). The chromatograph shows the nucleotide bases on the WT mESC (top) and the heterozygous base, highlighted in red, in the edited R1 mESC due to the base change (bottom). (B) The allelic ratio (AR) of *Pvt1* expression from F_1_ hybrid NPC clonal lines (purple) and edited R1 (R1-57) NPC clonal lines (pink) from targeted RNA-seq of *Pvt1*. Each dot represents a clonal line (*n* = 131 and *n* = 91, respectively). (C) Time-course plot of *Pvt1* AR in four different R1-57 NPC clonal lines over eight passages. The AR was measured using targeted RNA-seq. Each line on the plot is a clonal NPC line. (D) Allele-specific ATAC-seq and ChIP-seq of mESCs (green) and mE6 NPCs (blue). The top row shows SNP differences between the 129 allele and the CAST allele.

To determine whether *Pvt1* can achieve truly random monoallelic expression in the absence of genetic differences, we genetically edited a 129 × 129 R1 mESC to have a single base change on exon 9, allowing for distinction between wild-type (WT) and edited alleles (R1-57 line; Figure 4A). In contrast to the strong CAST bias in F_1_ hybrid NPC clonal lines, *Pvt1* in R1-57 NPC clonal lines showed no allelic bias, with either allele equally likely to be ME (Figure 4B). This fits the expected distribution for an aRME gene, indicating *Pvt1* ME is independent of parental origin (Figure S3A). A time-course experiment across passages demonstrated that the ME of *Pvt1* is constant in a non-hybrid line, confirming that the allelic choice is epigenetically maintained through cell division (Figure 4C). This observation suggests that the initiation and allelic choice of ME of *Pvt1* are part of a random stochastic process in the absence of genetic differences; however, genetic differences skew the stochastic process since most clones express *Pvt1* from the CAST allele rather than from the 129 allele. Close examination of the accessible regions at the *Pvt1* promoter reveals five SNP differences that overlap with differential ATAC signals between the two alleles, likely attributed to the CAST bias (Figure 4D).

### Differential binding of *Tfap2a* at the *Pvt1* promoter

**Figure 5.**
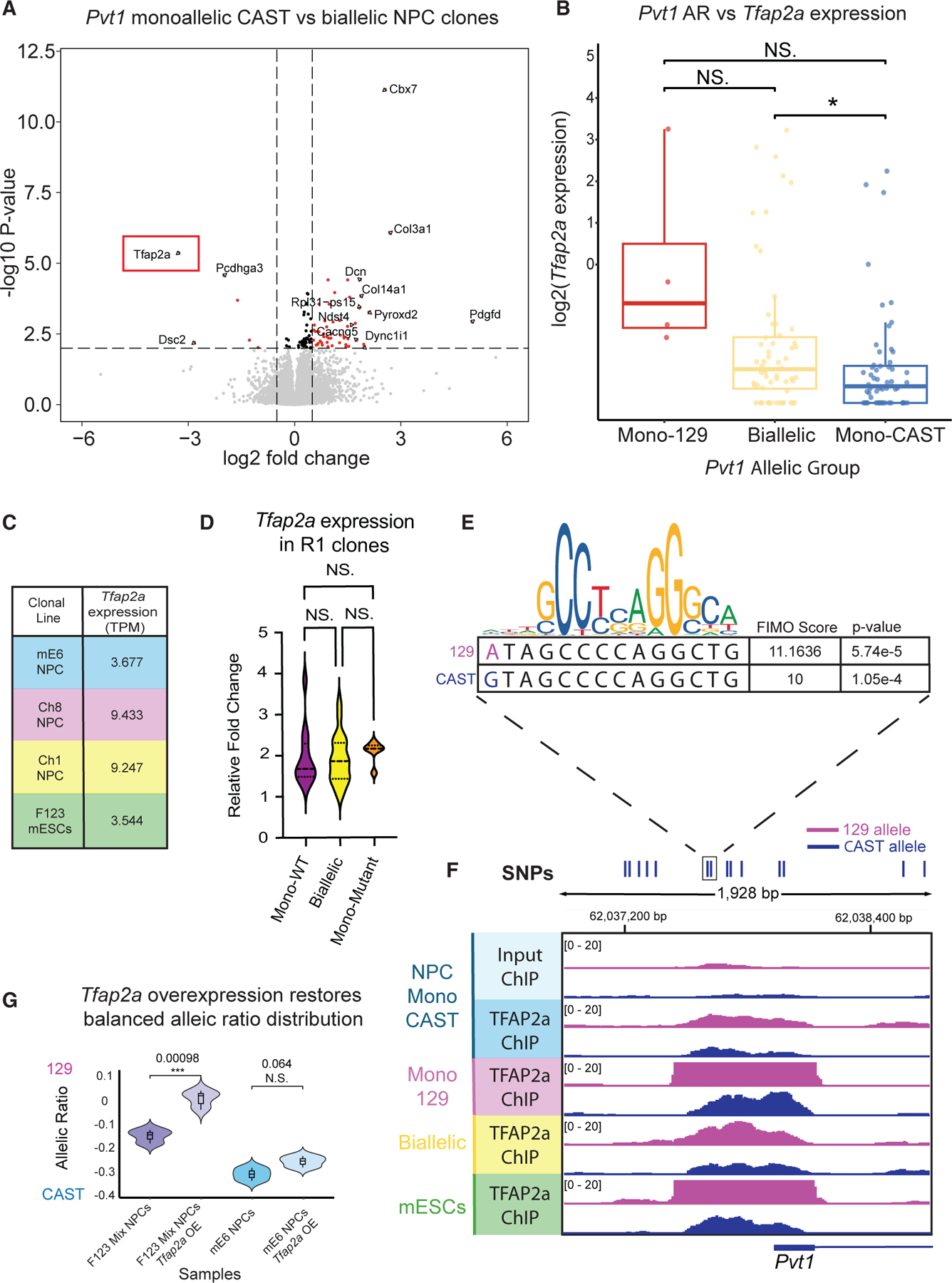
Differential binding of *Tfap2a* at the *Pvt1* promoter (A) Differential gene expression analysis (DESeq) between NPC clonal lines with *Pvt1* CAST monoallelic expression (*n* = 50) against NPC clonal lines with *Pvt1* biallelic expression (*n* = 58). Non-significant genes are in gray, and the significant genes based on log2 fold change and adjusted *p* value are in red, with the top 15 significant genes labeled. The adjusted *p* value cutoff is 0.01, and the log2 fold change cutoff is 0.5. *(B) Tfap2a* expression from F_1_-23 hybrid NPC clonal lines. *Pvt1* allelic expression status is based on an AR cutoff of 0.2 (*n* = 56, blue; *n* = 60, yellow; *n* = 4, red). Significance was calculated with a *t* test: NS *p* > 0.05 and **p ≤ 0.01. (C) Summary of *Tfap2a* expression levels in transcript per million (TPM) between the different F_1_-23 clonal lines. The color of the clonal line is based on *Pvt1* allelic status. *(D) Tfap2a* expression from RT-qPCR of R1-57 NPC clonal lines. *Pvt1* allelic expression status is based on an AR cutoff of 0.2 (*n* = 12 WT; *n* = 29, bi-allelic; *n* = 6, mutant). The relative fold change is compared to the NPC clonal line with the lowest *Tfap2a* expression. Significance was calculated with a *t* test: NS *p* > 0.05. (E) TFAP2a motif comparison between sequences from 129 allele (pink) and CAST allele (blue). (F) TFAP2a ChIP-seq tracks from four different samples: mE6 NPCs (blue), Ch8 NPCs (pink), Ch1 NPCs (yellow), and mESCs (green). The dotted line and black box around the SNPs demonstrate where the SNPs near the TFAP2a binding site can be found. (G) Violin plot of the AR from a non-clonal population of F_1_-23 NPCs (*n* = 3) and a non-clonal population of F_1_-23 NPCs with *Tfap2a* overexpression (*n* = 6, purple). Additionally, there is the AR of mE6 NPCs (*n* = 3) and mE6 NPCs with *Tfap2a* overexpression (*n* = 3, blue). The AR was obtained with targeted RNA-seq of *Pvt1*. *p* values were calculated with an unpaired *t* test: ***p ≤ 0.001. The experimental schematic is provided in Figure S4G.

We next sought to identify factor(s) that may be responsible for biasing the RME bias of *Pvt1* for the CAST versus the 129 genetic backgrounds. To this end, we performed differential gene expression analysis of *Pvt1* ME CAST clones versus *Pvt1* biallelic expressing clones. The analysis identified the transcription factor gene *Tfap2a*, a key transcriptional factor for the neural crest cell lineage^33^ (Figure 5A). In the F_1_ hybrid NPC clonal lines, *Pvt1* 129 ME clonal lines have higher *Tfap2a* expression than most *Pvt1* bi-allelic clones, and *Tfap2a* expression is lowest in *Pvt1* CAST ME clones (Figures 5B and 5C), indicating a negative correlation with *Pvt1* CAST expression. In R1-57 NPC clonal lines, *Tfap2a* expression was not different between clonal lines in the absence of genetic differences, further indicating that *Tfap2a* may underlie the genetic skew (Figure 5D).

Motif analysis at the *Pvt1* promoter showed a predicted Tfap2a binding site adjacent to an SNP (Figures 5E and S4A). FIMO^34^ (Find Individual Motif Occurences) analysis of the TFAP2a binding motif predicts stronger TFAP2a binding at the 129 promoter, although ME is skewed toward the CAST allele (Figure 5E). Based on an examination of other transcription factors that are predicted to occupy the *Pvt1* promoter overlapping or adjacent to SNPs (Figure S4A), neither *Six3* nor *NeuroG2* showed a significant difference in expression between the *Pvt1* monoallelic CAST and the bi-allelic clonal lines (Figures S4B and S4C).

TFAP2a ChIP in mESCs and NPCs confirmed preferential binding to the *Pvt1* promoter on the 129 allele over the CAST allele, as predicted by FIMO^34^ (Figure 5F). To assay whether the single SNP affects TFAP2a binding, we performed TFAP2a ChIP-qPCR on cells with plasmids with varying *Pvt1* promoters. ChIP-qPCR showed a significant increase in ChIP enrichment when the *Pvt1* CAST promoter was edited to have the 129 SNP mentioned in Figure 5E, compared to the CAST promoter without the edit and the 129 promoter edited with the CAST SNP (Figure S4F). The result demonstrates that the single SNP around the TFAP2a binding motif influences TFAP2a binding.

In mESCs, the 129 allele shows stronger H3K27ac and H3K4me3 signals at the Pvt1 promoter (Figures 3C and S3D), and endogenous tagging of the *Pvt1* promoter confirms that the 129 promoter is more transcriptionally active than the CAST promoter (Figures S4D and S4E). When TFAP2a expression is low, such as in *Pvt1* CAST ME clones (Figure 5C), binding events are low (Figure 5F), likely leading to the stochastic TFAP2a binding bias toward the inherently “stronger” 129 allele. Moreover, when *Tfap2a* expression is high, such as in the *Pvt1* 129 ME clonal line (Figure 5C), TFAP2a heavily occupies the 129 allele, but there is also increased binding on the CAST allele, suggesting a reduced sensitivity to the SNP-driven differences, making the allelic choice less biased (Figure 5F).

To further test our model, we sought to ask whether *Tfap2a* overexpression was sufficient to restore balance to the extent of RME in F_1_-23 hybrid NPCs. We transduced F_1_-23 mESCs with a doxycycline (dox)-inducible *Tfap2a* overexpression plasmid. Then, we added dox to the mESCs throughout differentiation to NPCs and to a stable NPC clonal line to answer whether increasing *Tfap2a* expression during the establishment of *Pvt1* ME and during the maintenance stage, respectively, can rescue the CAST bias (Figure S4G). The results show that in normal, non-clonal mixed NPCs, the AR is averaged out to around −0.15 due to the CAST skew. However, when *Tfap2a* is overexpressed, we can significantly alter the AR distribution toward an AR of 0, indicating that the CAST skew is no longer present (Figure 5G). There is less of a change to the AR when *Tfap2a* is overexpressed in a stable NPC clonal line, demonstrating that differences in TFAP2a binding have the strongest effect during the initiation and establishment stage of aRME.

In summary, the initiation and allelic choice of *Pvt1* aRME are part of a random stochastic process. However, in our hybrid line, this process is skewed due to genetic SNP differences, leading to differential binding of TFAP2a and possibly other factors at the promoter. Our data from the hybrid line also suggest a two-step model for aRME establishment at *Pvt1*. When the *Tfap2a* expression is low, there is a sensitivity to the SNP, and the preferred allele, in our case, the 129 allele, is likely transcriptionally activated first and then gets silenced, causing the genetic skew of *Pvt1* ME toward the CAST allele. However, when there is high expression of *Tfap2a*, there is less sensitivity to the SNP and a loss of the genetic bias as indicated by the overexpression assay.

### *Pvt1* monoallelic expression regulates cell growth

**Figure 6.**
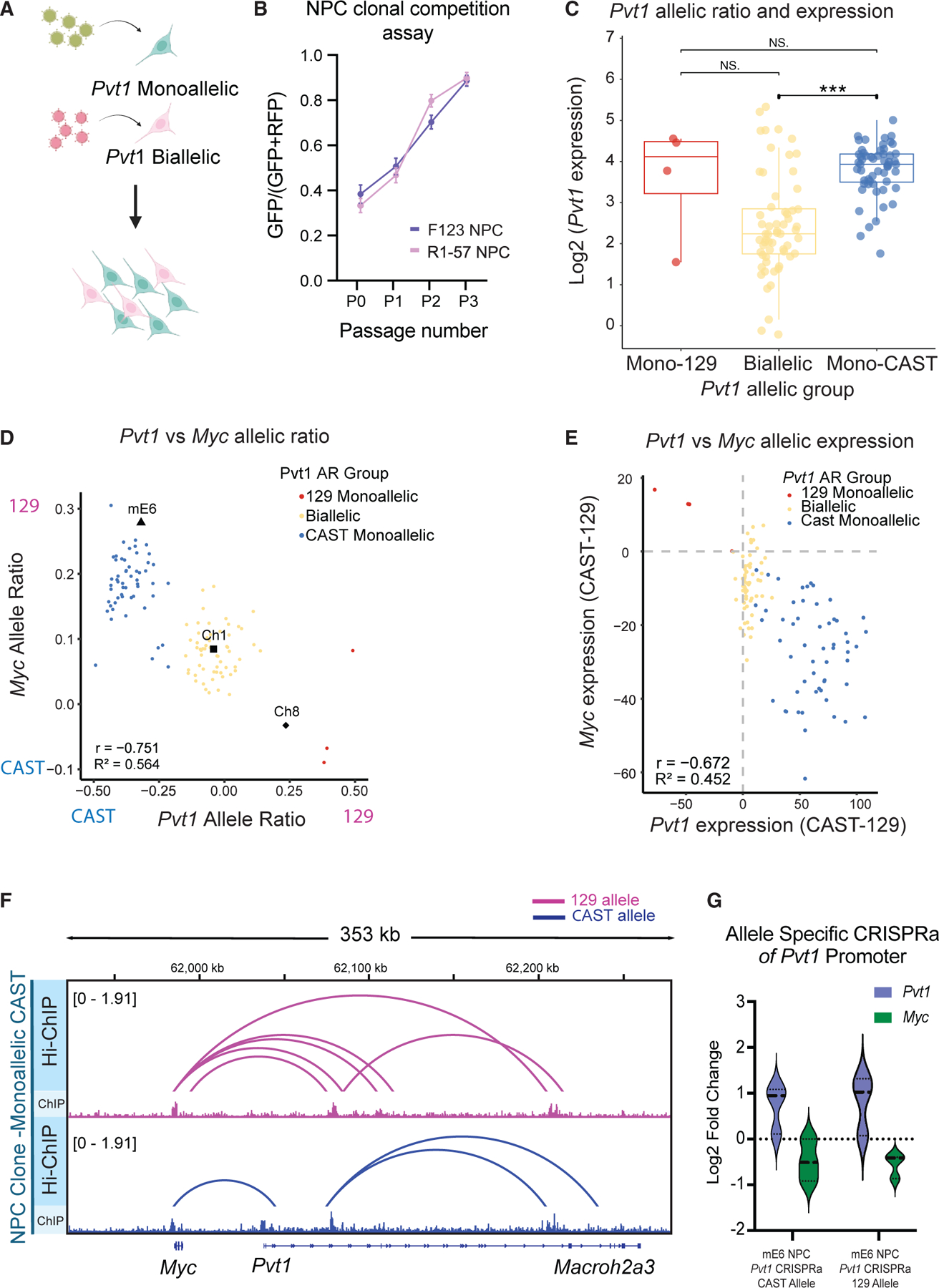
*Pvt1* monoallelic expression regulates cell growth (A) Experimental schematic of competition assay of NPC clonal lines based on *Pvt1* allelic status. (B) Quantification of competition assay from Figure 5A for two groups of clonal lines: clonal lines from F_1_-23 NPCs and clonal lines from R1-57 NPCs. The GFP/RFP ratio is quantified by the number of cells with GFP divided by the total number of cells expressing both GFP and RFP. Each dot and error bar represents the mean ratio and standard deviation for all clonal line combinations in each group for each passage number. (C) Plot comparing *Pvt1* allelic group versus *Pvt1* expression. The *Pvt1* allelic expression group was based on an AR cutoff of 0.2 (*n* = 56, blue; *n* = 60, yellow; *n* = 4, red). Significance was calculated with a *t* test: NS *p* > 0.05 and ***p ≤ 0.001. *(D) Pvt1* allelic ratio (AR) versus *Myc* AR from F1-23 NPC clonal lines. The clonal lines are grouped based on *Pvt1* allelic expression status. R^2^ is the calculated coefficient of determination, and r is the correlation coefficient. Each dot represents a clonal line (*n* = 119). *(E) Pvt1* expression (CAST TPMs − 129 TPMs) against *Myc* expression (CAST TPMs − 129 TPMs). The clonal lines are grouped based on *Pvt1* allelic expression status. (F) Independent H3K27ac Hi-ChIP (blue) and H3K27ac ChIP-seq (light blue) of F_1_-23 hybrid NPC clonal line with *Pvt1* expressed from the CAST allele. *p* < 0.1 significant loops called from Hi-ChIP data. (G) qPCR plot of log2 fold change for CRISPR activation (CRISPRa) experiment in mE6 NPCs. The two samples are CRISPRa guides targeted with the *Pvt1* CAST promoter and the *Pvt1* 129 promoter (*n* = 3 each). Quantification of the fold change in *Pvt1* expression is in blue and in *Myc* expression is in green. The dotted line marks the baseline from mE6 WT cells.

To evaluate the consequences of *Pvt1* monoallelic expression, we performed a competition assay between *Pvt1* ME clones and *Pvt1* bi-allelic clones using fluorescent markers (Figure 6A). *Pvt1* ME clones outcompeted *Pvt1* bi-allelic clones over time, highlighting a growth advantage phenotype when *Pvt1* is monoallelically expressed, independent of genetic differences (Figure 6B). Interestingly, when comparing the *Pvt1* expression and AR of Pvt1 in individual clonal NPC lines, we observed increased expression of *Pvt1* in monoallelic clones compared to bi-allelic clones (Figure 6C), thereby inverting the expected relationship between gene copy number and expression level. The same trend was observed in non-hybrid R1-57 lines (Figure S5A), indicating that the effect is an intrinsic property of the gene independent of genetic differences.

Next, we explore if this growth advantage is due to interactions with Myc, a neighboring bi-allelically expressed gene (Figures 1E and S1D). *Myc* expression levels showed no significant difference between hybrid and non-hybrid clonal lines (Figures S5B and S5C). However, comparing the *Pvt1* AR against the *Myc* AR across all NPC clones revealed an inverse relationship between their ARs in each clonal line (Figure 6D), suggesting an inhibitory effect in *cis* of *Pvt1* ME on *Myc* expression. We reached the same conclusion when we looked at the *Pvt1* expression compared to the *Myc* expression in an allele-specific manner (Figure 6E), highlighting the importance of conducting allele-specific analysis to detect this regulation.

To further investigate the regulation in *cis* between *Pvt1* and *Myc*, we performed allele-specific H3K27ac HiChIP, a protein-directed method to capture chromosome conformation, and H3K27ac ChIP-seq to investigate the 3D enhancer connectomes between the two alleles (Figure 6F). When the *Pvt1* promoter is active, *Myc* principally contacts the *Pvt1* promoter, and *Pvt1* contacts *Myc*. However, on the *Pvt1*-inactive allele, *Myc* gains contacts with at least four intragenic enhancers within the introns of *Pvt1*, demonstrating the enhancer-promoter competition within the locus. Allele-specific CRISPR activation of the *Pvt1* promoter in monoallelically expressing clonal lines resulted in an increase in *Pvt1* expression and a decrease in *Myc* expression on the targeted allele (Figure 6G), confirming the *cis*-regulatory effect. These findings together suggest that the monoallelic accessibility of *Pvt1* adds an additional layer of gene regulation to the *Pvt1-Myc* locus in early development. By unraveling the dynamics between *Pvt1* monoallelic expression and *Myc*, we provide insight into how RME genes may serve as regulatory controls within their locus to drive different early differentiation programs.

Lastly, we observed that not all aRME genes behave like *Pvt1*. Genes such as *Cbr3*, *Eid1*, and *Sox1* exhibit increased expresion bi-allelic versus monoallelic expressing clones, with *Sox1* displaying genetic effects (Figures S5D, S5E, and S5H). On the other hand, there are aRME genes, such as *Mettl5* and *Neat1*, that resemble *Pvt1*, with higher expression in ME versus bi-allelic clones, with *Neat1* also displaying genetic effects (Figures S5F and S5G). With these data, we demonstrate how ME can affect both gene expression and locus regulation, such as *Pvt1-Myc*, to underscore the diverse functional roles for aRME genes in early development.

## DISCUSSION

In this study, we analyzed 120 NPC clonal lines to unbiasedly identify genes that exhibit diverse ME patterns in NPCs. The large dataset of allele-specific RNA-seq data allowed us to correlate ARs with gene expression and the expression levels of genes with which they may interact. Comprehensive epigenomic profiling of three NPC clonal lines reveals epigenetic marks at aRME genes, providing a foundation to further explore ME mechanisms and consequences.

Focusing on *Pvt1*, we leveraged the dataset to investigate *Pvt1* ARs, expression, and interactions with other genes, distinguishing genetic from epigenetic contributions to *Pvt1* ME in NPCs. We previously showed that *Pvt1* undergoes aRME,^26^ but more extensive clone analysis identified a CAST bias in *Pvt1* ME and revealed that genetic differences can skew what would otherwise be a random stochastic process. These datasets set the stage for deeper exploration of aRME genes, their mechanisms, and their impacts.

Mechanistically, our data support a model where, when expression of *Tfap2a* is low and binding is infrequent, there is a biased stochastic process involved in *Pvt1* allelic choice, where the initial active allele is silenced through differentiation. However, when the expression of *Tfap2a* is high and the *Pvt1* promoter occupancy is high, the stochastic process is no longer biased. The initiation and choice of aRME have been thought to be part of a truly random process driven by a stochastic process; however, we show that the contributions of genetic differences cannot be ignored in this model. In the case of *Pvt1*, differences in transcription factor binding can skew the stochastic process in a hybrid line. In future studies, it will be interesting to look at how common genetic variation modulates aRME genes and influences key biological processes.

We found that roughly 6% of genes show autosomal monoallelic expression, while 2.7% of genes exhibit aRME; however, the functional consequences of aRME are rarely known. It is likely that aRME genes play a key role in development since the number of genes that are RME increase with differentiation^14^ and can create cellular heterogeneity by being variably monoallelic or bi-allelic. In the case of *Pvt1*, having ME results in a clear growth phenotype, which creates cellular heterogeneity in precursor cells, such as NPCs, as they begin to differentiate into different neuronal lineages. Interestingly, the observed growth phenotype is likely to come from two sources: (1) an increase in Myc expression in *cis* due to enhancer-promoter competition and (2) the transcriptional over-compensation of *Pvt1* expression in monoallelically expressing clones. *Pvt1* has been shown in both developmental and cancer contexts to promote proliferation.^20,35^

Moreover, work on aRME genes has mainly focused on H3K4me3, H3K9me3, and DNA methylation to understand the epigenetic maintenance of monoallelic expression^6,14–16^; however, we show the *Pvt1* promoter to be bivalently marked by both H3K4me3 and H2AK199ub, in addition to the other common marks. In mESCs, the promoter is also marked by both H3K4me3 and H3K27me3. Bivalent promoters are known to mark developmental and lineage-specific genes^31^ to allow for quick transitions, supporting the hypothesis that aRME genes play a role in cell differentiation and identity^13,14^ in early differentiations. In the cancer context, developmental genes with bivalent promoters are known to be targets of aberrant hypermethylation due to a loss of H3K4me3.^36–38^ In the case of *Pvt1*, understanding the epigenetic landscape during early development provides insight into how *Pvt1* becomes dysregulated in adult human cancers. This acts as a double-edged sword where bivalency and ME are advantageous in early development but make *Pvt1* more susceptible to methylation in adult cancers. *Pvt1* is a strong example of how the epigenetic landscape from ME in early development can lead a locus to be vulnerable to aberrant methylation later in adulthood.^36^

While we detail here the genetic and epigenetic mechanisms of *Pvt1* ME and its functional consequence, there are several aME genes from our list that are associated with neurodevelopmental disorders,^39^ such as *Grik3*, *Mettl5*, *Gap43*, and *Tubb3*. Additionally, there are several aME genes whose dysregulation is associated with cancers when misregulated, such as *Mmp14*, *Nid1*, *Mdm2*, *Cd36*, and *Id3*. Further investigation of these genes can provide valuable information on the functional consequences of ME in development and cancer. Additionally, our data showing the effects of genetic variation of aRME lay the foundation for future studies to dive deeper into how natural genetic variation affects aRME genes and may impact brain development, neurodevelopmental disorders, and cancer.

### Limitations of the study

This study relies on clonal NPC lines derived from hybrid mESCs, which may not fully recapitulate *in vivo* developmental processes. Additionally, bulk RNA-seq and ChIP-seq approaches, while powerful, average signals across cells and may obscure single-cell variability or transient states. While we illuminated the mechanisms of *Pvt1* aRME, this may not be generalizable to other aRME genes. Further investigation is needed to create a mechanistic model for aRME genes in general. Moreover, incorporating *in vivo* models and single-cell data would further strengthen the conclusions we have made in this study.

## RESOURCE AVAILABILITY

### Lead contact

Further information and reagent requests should be directed to and fulfilled by the lead contact, Howard Y. Chang (howchang@stanford.edu).

### Materials availability

Cell lines and reagents are available upon request.

## STAR★METHODS

### EXPERIMENTAL MODEL AND STUDY PARTICIPANT DETAILS

#### Cell culture

F_1_-23 (male) and F_1_-21.6 (female) hybrid mESCs (CAST x 129), a gift from Edith Heard’s lab, and R1 mESCs (ATCC, SCRC-1011) were grown on 0.1% gelatin (StemCell Tech, 7903) covered plates with MEFs (Applied Stem Cell, ASF-1216), 1000 units/mL LIF (Millipore, ESG1107), and FBS (Thermo, A5670402). mESCs were then transitioned into N2B27 medium with 2i (1uM PD (Tocris, 4192) and 5uM CHIR (Tocris, 4423)) + 1000 units/mL LIF conditions with 0.1% gelatin-coated plates. N2B27 medium contains 1:1 DMEM (Gibco, 11995073) and Neurobasal medium (Gibco, 21103-049), 1x N2 (Invitrogen, 17502-048), 0.5x B27 (Invitrogen, 17504-044), 1x Glutamax (Thermo, 35050061), 50mM Beta-Mercaptoethanol (Invitrogen, 21985-023), and 1x Pen/Strep (Thermo, 15140163). For NPC differentiation, mESCs in 2i+Lif were plated onto a 0.1% gelatin-coated plate with N2B27 medium for 7 days. Cells were then dissociated with accutase (Millipore, SCR005) and placed into plates with N2B27 medium with 10ng/mL EGF (Peprotech, 315-09) and 10ng/mL FGF (R&D Systems, 3139-FB-025) for 3 days to form neurospheres. Neurospheres were plated onto gelatinized plates to allow for the migration of NPCs out of the neurospheres with N2B27 medium with EGF and FGF. NPCs were passaged once before single cells were subcloned to create NPC clonal lines.

### METHOD DETAILS

#### Cell line generation

##### R1-57 mESC

R1 mESCs were edited using nucleofection of Cas9 RNP with a single crRNA (atcacctcataggttcaaca) and a homologous donor block from IDT that contains the single base change on Exon 9 of *Pvt1*, which also destroys the PAM site. mESCs were then screened using genomic PCR followed by sanger sequencing to isolate the mESC clone with the correct edit.

##### RNA-seq

RNA was extracted using Trizol (Thermo, 15596026) and purified using Zymo Direct-Zol RNA miniprep (Zymo, R2052). RNA from female (F_1_-21.6) NPC clonal lines was gifted from Edith Heard’s lab.^16^ RNA samples were then sent to Illumina for directional mRNA library prep with poly A enrichment. Reads were sequenced 150 ×150 on the NovaSeq 600 platform for 40M paired reads per sample.

##### ChIP-seq

Cells are fixed with 1% PFA (Fisher, 50980487) for 10 min at room temperature, then quenched for 5 min with 1.25M glycine (Fisher, BP381-500) to 125mM final concentration. Cells were washed with PBS and pelleted with centrifugation for 5 min at 4C. Cells were resuspended in lysis buffer 1 (50mM Hepes-KOH pH 7.5 (Life Technologies, 15630-080), 140mM NaCl (Thermo, AM9760G), 1mM EDTA (Invitrogen, AM9260G), 10% Glycerol, 0.5% NP-40 (Millipore, 492016), 0.25% Triton X-100 (Sigma, 93443), and protease inhibitor (Roche, 11873580001) and incubated for 10 min at 4C. Cells were then centrifuged for 5 min at 4C. Cells were then resuspended in lysis buffer 2 (10mM Tris-HCl pH 8.0 (Invitrogen, 15568-025), 200mM NaCl, 1mM EDTA, 0.5mM EGTA (Thermo, 50-255-956), and protease inhibitor) and incubated for 10 min at 4C followed by centrifugation. Cells were then resuspended in lysis buffer 3 (10mM Tris-HCl pH 8.0, 100mM NaCl, 1mM EDTA, 0.5mM EGTA, 0.1% Na-Deoxycholate (Bioworld, 40430018-2), 0.5% N-Laroylsarcosine (Sigma, L7414), and protease inhibitor). Cells were then sonicated in a Covaris E220 for 18 min at Fill level 10, Duty Cycle 5, PIP 140, and Cycles/Burst 200. A small portion of sonicated chromatin is then de-crosslinked, treated with proteinase K (Thermo, 25-530-049) and RNase A (Invitrogen, 12-091-021), purified, and quantified with Qubit DNA HS (Thermo, Q32854). Sonicated chromatin is then divided into tubes with 12.5 μg or 25ug of sonicated chromatin per histone or TF ChIP sample respectively. Sonicated chromatin is then incubated with 5ug or 7.5ug of antibody for histone or transcription factor ChIP, respectively, overnight at 4C. 100ul of Protein A/G (Thermo, 26162) beads per sample are washed with 0.5% BSA block solution and incubated with sonicated chromatin for 6 h at 4C. Beads are then washed with 5 times with RIPA buffer (50mM HEPES-KOH, 1mM EDTA, 1% NP-40, 0.7% NaDeoxycholate, 500mM LiCl, and protease inhibitor). Beads are then eluted with elution buffer (50mM Tris-HCl, 10mM EDTA, and 1% SDS) at 65C for 30 min. Samples are then de-crosslinked, treated with proteinase K and RNase A, and purified using Zymo ChIP Clean and Concentrate (Zymo, D5205). 10-20ng of ChIP DNA is then used to create libraries using NEBNext Ultra II DNA Library Prep Kit (NEB, E7645S). Libraries were sequenced on NextSeq or NovaSeq X Plus with PE 150 × 150 for 100million reads per sample. Antibodies used: H3K27ac (Abcam, 4729), H3K27ac (Active Motif, 39133), H3K4me2 (Abcam, ab7766), H3K4me3 (Active Motif, 39159), H3K9me3 (Abcam, ab8898), H2AK199ub (Cell signaling, D27C4), H3K27me3 (Cell Signaling, C36B11), RNA Pol II-RPB1 (Bio-legend, 664906), and TFAP2a (Novus, NB100-74359). All samples have biological replicates.

##### Bisulfite-seq

DNA was extracted using genomic lysis buffer (100mM Tris-HCl pH 8.0, 50mM EDTA, and 1% SDS), incubated with Proteinase K (Thermo, 25-530-049), and purified using phenol-chloroform (Thermo, 15-593-031) followed by isopropanol precipitation. Library was then prepared using TruSeq Methyl Capture EPIC library prep kit (Illumina, FC-151-1002). Library was then sequenced on the NextSeq 500/500 with paired end reads 150 × 150 bp with 200 million reads per sample.

##### Targeted RNA-seq

RT-qPCR with 50ng of RNA per reaction, done in triplicates, with primers for target gene from Table S4 and using Brilliant II SYBR Green qRT-PCR 1-step Master Mix (Agilent, 600825). The qPCR product is diluted 1:20 and 1ul is used for nested PCR with adapter primers. PCR product is then diluted 1:20 and 1ul is used for library indexing PCR. Indexed PCRs are then pooled together and quantified using NEBNext library quantification (NEB, E7630L). Library is then sequenced on the MiSeq at 75 × 75 bp paired end with 40% PhiX.

##### Hi-ChIP

HiChIP was performed as previously described^53^ with minor adjustments. 10 million cells were crosslinked at a volume of 1mL of 1% formaldehyde (Fisher, 50980487) per 1 million cells for 10 min at room temperature and quenched with 1.25M glycine (Fisher, BP381-500) for final concentration of 125mM. Cells were then washed with PBS. Cells are lysed to isolate chromatin. The chromatin is then digested with MboI restriction enzyme (NEB, R0147M), followed by end-pair with Klenow (NEB, M0212L) and biotinylated-dATP (Jena, NU-835-BIO14-L), ligation with T4 ligase (NEB, M0202L), and sonication with a Covaris for 4 min. Sonicated and ligated chromatin is then incubated with H3K27ac (Active Motif, 39133) antibody overnight at 4C. Antibody bound chromatin is then captured with Protein A beads (Thermo, 26162), washed, and eluted from the beads. ChIP DNA then undergoes biotin pulldown with Streptavidin C1- beads (Invitrogen, 65001) followed by Tn5 tagmentation for library generation. Libraries are sequenced on Illumina HiSeq 4000 for 200 million paired-end reads at 150 × 150 bp per sample.

##### RT-qPCR

50ng of RNA per reaction, done in triplicates, with primers from Table S4 and using Brilliant II SYBR Green qRT-PCR 1-step Master Mix (Agilent, 600825).

#### Competition assay

*Pvt1*-monoallelic or *Pvt1*-biallelic NPC clonal lines are transduced with GFP or RFP expressing lentivirus, respectively, with polybrene (Millipore, TR-1003-G) containing media. After 48 h, GFP or RFP positive cells undergo selection with puromycin (Thermo, A1113803). For the assay, 30,000 cells of GFP positive *Pvt1* monoallelic cells are plated with 30,000 cells of RFP positive *Pvt1* biallelic cells in a 48-well plate. There were two groups of clonal lines: clonal lines from F1-23 NPCs and clonal lines from R1-57 NPCs. For F_1_23 NPCs, there are 5 different clonal line combinations with two biological replicates each. For R1-57 NPCs, there are 2 different combinations with two biological replicates each. All combinations were done in technical triplicates. Fluorescent was measure with flow cytometry using an Attune NxT at passage 0, and after every passage. Cells were passage 1:2 every other day. GFP ratio was calculated by dividing the number of cells with GFP signal over the total number of cells with either GFP or RFP fluorescence.

#### Plasmid ChIP-qPCR

Plasmids were cloned using HiFi assembly mix from NEB. PCR was used to amplify fragments of interest from genomic DNA and generic plasmid with red fluorescent protein. Primers are found in Table S4 For single base change at the promoter, plasmids with the unmodified CAST and 129 Pvt1 promoter were digested with Bsu36I and HindIII. The plasmid was then reassembled with PCR products where the primers used contained the base change.

For the experiment, 3ug of the plasmid DNA of choice was transiently transfected into a 60% confluent 10cm plate of NPCs using Lipofectamine 3000 (Invitrogen, L3000008). Cells were then incubated for 24 h and harvested for TFAP2a ChIP as previously described. qPCR was then performed for the *Pvt1* promoter region, RFP, and a negative control region for the samples and their input. For the calculations, Ct values for the input was normalized by subtracting 4.32 cycles since it was a 5% input. Then the sample was normalized by subtracting the RFP Ct value from the input. Fold enrichment over input is then calculated from log2.

#### Tfap2a overexpression

Lentivirus was produced using HEK293T but transiently transfecting a 6 well of cells with 2ug of pLV-teto-Tfap2a (Addgene,70274)^54^ modified to have puromycin resistance, 1ug of pMD2.G, and 2ug of psPax2 (2ug) with lipofectamine 3000. Media was changed after 12 h. Viral supernatant was collected after 48 h and 72 h. Virus was concentrated using 4x Lenti-X concentrator according to manufacturer’s protocol.

A 6-well plate of F_1_-23 mESCs or mE6 NPCs were then transduced with 10ul on concentrated virus with polybrene. Media was changed after 24 h and puromycin selection was carried out for 1 week. F_1_-23 then began differentiation to NPCs with the addition of doxycycline with each media change. mE6 NPCs were also treated with doxycycline for three passages before RNA was harvested. RNA was harvested from all the samples (*n* = 3, biological replicates) and allelic ratio was quantified using Targeted RNA-seq as mentioned previous but followed by sanger sequencing.

#### CRISPR activation

Stable NPC cell lines with generated using a CRISPRai system previously published from the lab.^55^ Virus was produced as mentioned above with Sa-gRNA scaffold plasmid from the lab^55^ with gRNA sequences in Table S4. HEK293T were transfected with a pool of 3 gRNA plasmids per target for virus production. Stable CRISPRai cells were then transduced with the virus. Media was changed after 24 h. Cells then underwent zeocin selection for 1 week.

Cells with gRNA and CRISPRai system were then seeded to 30% confluency and doxycycline. Cells were grown out with doxycycline for 72 h before RNA was harvested for qPCR quantification for *Pvt1* and *Myc* expression.

#### Custom N-masked genome

Custom N-masked genome is created using SNPsplit_genome_preparation from SNPsplit^41^ (v0.5.0) using a reference mouse genome (mm10) and VCF file containing SNP information for mm10 from the mouse genome project^56^ (mgp.v5.merged.snps_all.dbSNP142.vcf). The –dual hybrid mode is used with strain1 set to 129S1/SvlmJ and strain 2 set to CAST/EiJ. N-masked genome is used for all downstream alignments for genomic analysis. This also created an SNP-file containing all SNPs between the two strains that is used downstream to split reads on informative SNPs. Reads without informative SNPs are not considered in allele-specific analysis.

#### RNA-seq analysis

A portion of the female NPC clonal RNA-seq fastqs were extracted from GSE259400.^40^ Reads are aligned with STAR^42^ (2.7.10) with default parameters using a genome index created with a custom N-masked genome and Gencode vM25 GTF file. Aligned reads are then split based on informative SNPs using SNPsplit^41^ (V0.5.0) using –paired mode. Transcripts are quantified from non-SNP-split and SNP-split aligned reads using Salmon^43^ (v1.10.0) using a custom gentrome, (genome transcriptome) with an N-masked genome and gencode vM25 GTF file, and the –validateMappings option. Tximport^44^ (v1.34.0) is then used on allele-specific and non-allele specific Salmon abundance files to convert the transcripts to gene-level abundance and counts for downstream analysis.

Using the tximport generated matrix, the allele-specific counts is used to calculate allelic ratio by dividing 129 read counts over the total counts of 129 and CAST reads minus 0.5 ((129/(CAST +129))-0.5). Calculating with allelic ratio with abundance instead of counts gave similar results. Allelic ratio is calculated for each gene for each sample (NPC clonal line). Mean of allelic ratio is calculated by averaging the allelic ratio for every gene across all female and male NPC clonal lines. Variance is calculated across both female and male NPC clonal lines. There are 6 groups of genes: Biallelic (green), X-linked (brown), imprinted (purple), random monoallelic (RME) genes (orange), 129-biased monoallelic genes (red), and CAST-biased monoallelic genes (blue).

Differential gene expression and principal component analysis was done with DESeq2^45^ (v1.46.0) on non-allele-specific counts from tximport matrix. Total gene-expression of each sample is extracted from non-allele specific normalized expression from DESeq2 and allele-specific gene expression is extracted from abundance from allele-specific tximport matrix. F123 NPC clonal lines were grouped based on *Pvt* allelic status using allelic ratio guideline of *Pvt1* Monoallelic cast if AR > 0.2, *Pvt1* Biallelic if AR < 0.2 or AR > −0.2, and *Pvt1* Monoallelic 129 if AR < −0.25. Allelic ratio cutoff was chosen based on the distribution of *Pvt1* AR across all NPC clones. R1-57 NPC clonal lines were grouped based on *Pvt*1 allelic status using allelic ratio guideline of *Pvt1* Monoallelic WT allele if AR > 0.2 etc.

#### ChIP-seq analysis

Reads are trimmed with trim-galore^46^ (v0.5.0) and aligned with an custom N-masked genome using Bowtie2^47^ (v2.3.4.1). Aligned reads are then deduplicated with samtools (v1.16.1) markdup and indexed. Aligned reads are then split based on informative SNPs with SNPsplit^41^ (V0.5.0) with –paired option. Coverage tracks are then created using deeptools^48^(v3.3.1) bamCoverage using non-allele-specific and allele-specific bam files that are normalized using RPGC and bin size of 10. Matrix used for the heatmaps were created using deeptools^48^(v3.3.1) computeMatrix using Bigwig files and heatmaps were generated using plotHeatmap.

#### Motif analysis

Fasta files were created with ∼500bp sequences of the *Pvt1* promoter from the 129-allele and the CAST-allele. FIMO from the MEME suite^34,49^ is then used to search the fasta sequences for occurrences for the longest TFAP2A motif sequence (ID: MA0003.2) to ensure the base with the SNP difference is include in the analysis. Motif sequence was collected from JASPAR database.^57^

#### ATAC-seq analysis

ATAC-seq data for NPC clonal lines from were downloaded from GSE84646.^15^ Fastq files were trimmed, aligned, and deduplicated as mentioned above for ChIP-seq analysis. Reads were then split on informative SNPs as mentioned in ChIP-analysis. Coverage tracks are then created using deeptools^48^(v3.3.1) bamCoverage using non-allele-specific and allele-specific bam files that are normalized using CPM and bin size of 50.

#### Bisulfite-seq analysis

Fastq files were aligned using Bismark^50^ (v0.22.3) using a genome prepared by bismark_genome_preparation with Bowtie2^55^ (v2.3.4.1) and the N-masked genome. Aligned reads were then deduplicated using deduplicate_bismark. Aligned reads are then split based on informative SNPs with SNPsplit^41^ (V0.5.0) with – bisulfite and – paired option. Coverage tracks are created using deeptools^48^(v3.3.1) bamCoverage using allele-specific bam files that are normalized using CPM and bin size of 50.

#### Hi-ChIP analysis

HiChIP fastq files were first passed through the HiC-Pro^51^ pipeline with for mapping with Bowtie2^47^ using the N-masked genome with minimum mapping quality set to 10, and set to remove singleton, multi-mapped, and duplicated reads. Pipeline is then set in allele-specific mode and reads are split based on informative SNPs. Reads are then assigned to MboI restriction enzyme, separating the valid pairs from invalid ligation products. This is use as quality check for the library. We only consider libraries that have greater than 85% valid pairs in our libraries. Reads are filtered to dangling end, self-circle pairs, and duplicated valid pairs. Allele-specific valid pairs are then used to create raw contact maps with resolution 10,000 and corrected using ICE normalization. Loop are called using FitHiChIP^52^ (v11.0) using the configuration file stringent FitHiChIP model with coverage bias regression (configfile_P2P_Biascorrection_CoverageBias).

## Supplementary Material

1

2

3

4

5

Supplemental information can be found online at https://doi.org/10.1016/j.celrep.2025.116554.

## Figures and Tables

**Table:** KEY RESOURCES TABLE

REAGENT or RESOURCE	SOURCE	IDENTIFIER
Antibodies		
H3K27ac	Abcam	Cat# 4729; RRID: AB_2118291
H3K27ac	Active Motif	Cat# 39133; RRID: AB_2561016
H3K4me2	Abcam	Cat# ab7766; RRID: AB_2560996
H3K4me3	Active Motif	Cat# 39159; RRID: AB_2615077
H3K9me3	Abcam	Cat# ab8898; RRID: AB_306848
H2AK199ub	Cell signaling	Cat# D27C4
H3K27me3	Cell Signaling	Cat # C36B11
RNA Pol II-RPB1	Biolegend	Cat# 664906; RRID: AB_2565554
TFAP2a	Novus	Cat# NB100-74359; RRID: AB_1048155

Bacterial and virus strains		

NEB^®^ 5-alpha Competent E. coli	New England Biolabs (NEB)	C2987H

Chemicals, peptides, and recombinant proteins		

0.1% gelatin	StemCell Tech	7903
LIF	Millipore	ESG1107
FBS	Thermo Scientific	A5670402
PD	Tocris	4192
CHIR	Tocris	4423
DMEM	Gibco	1995073
Neurobasal medium	Gibco	21103-049
N2 Supplement	Invitrogen	17502-048
B27 Supplement	Invitrogen	17504-044
Glutamax	Thermo Fisher Scientific	35050061
Beta-Mercaptoethanol	Invitrogen	21985-023
Pen/Strep	Thermo Fisher Scientific	15140163
accutase	Millipore	SCR005
EGF	Peprotech	315-09
EGF	R&D Systems	3139-FB-025
Trizol	Thermo Fisher Scientific	15596026
Zymo Direct-Zol RNA miniprep	Zymo	R2052
PFA	Fisher Scientific	50980487
glycine	Fisher Scientific	BP381-500
Hepes-KOH pH 7.5	Life Technologies	15630-080
NaCl	Thermo Fisher Scientific	AM9760G
EDTA	Invitrogen	15568-025
NP-40	Millipore	492016
Triton X-100	Sigma	93443
protease inhibitor	Roche	11873580001
Tris-HCl pH 8.0	Invitrogen	15568-025
EGTA	Thermo Fisher Scientific	50-255-956
Na-Deoxycholate	Bioworld	40430018-2
N-Laroylsarcosine	Sigma	L7414
proteinase K	Thermo Fisher Scientific	25-530-049
RNase A	Invitrogen	12-091-021
Qubit DNA HS	Thermo Fisher Scientific	Q32854
Protein A/G	Thermo Fisher Scientific	26162
Zymo ChIP Clean and Concentrate	Zymo	D5205
NEBNext Ultra II DNA Library Prep Kit	NEB	E7645S
TruSeq Methyl Capture EPIC library prep kit	Illumina	FC-151-1002
Brilliant II SYBR Green qRT-PCR 1-step Master Mix	Agilent	600825
NEBNext library quantification	NEB	E7630L
MboI restriction enzyme	NEB	R0147M
Klenow	NEB	M0212L
T4 ligase	NEB	M0202L
biotinylated-dATP	Jena Bioscience	NU-835-BIO14-L
Protein A beads	Thermo Fisher Scientific	26162
Streptavidin C1- beads	Invitrogen	65001
polybrene	Millipore	TR-1003-G
puromycin	Thermo Fisher Scientific	A1113803
Lipofectamine 3000	Invitrogen	L3000008

Deposited data		

Bisulfite sequencing	This paper	GSE287455
ChIP Sequencing	This paper	GSE287456
Hi-ChIP sequencing	This paper	GSE287457
RNA sequencing	This paper	GSE287458
RNA-sequencing of female NPCs	Hauth et al.^40^	GSE259400

Experimental models: Cell lines		

F_1_-26 female hybrid mESCs	Gendrel et al.^6^	NA
F_1_-23 male hybrid mESCs	Gendrel et al.^6^	NA
R1 MESCs	ATCC	SCRC-1011
HEK293T	ATCC	CRL-3216
Mouse embryonic fibroblast (MEFs)	Applied Stem Cell	ASF-1216

Recombinant DNA		

pLV-teto-Tfap2a	Addgene	70274

Software and algorithms		

SNPsplit	Kruger and Andrews.^41^	v0.5.0
STAR	Dobin et al.^42^	2.7.10
Salmon	Patro et al.^43^	v1.10.0
Tximport	Soneson et al.^44^	v1.34.0
DESeq2	Love et al.^45^	v1.46.0
trim-galore	Martin et al.^46^	v0.5.0
Bowtie2	Langmead et al.^47^	v2.3.4.1
deeptools	Ramirez et al.^48^	v3.3.1
MEME suite	Bailey et al.^49^	4.11.3
Bismark	Krueger et al.^50^	v0.22.3
HiC-Pro	Servant et al.^51^	v2.10.0
FitHiChIP	Bhattacharyya et al.^52^	v11.0

## Data Availability

Bisulfite sequencing, Hi-ChIP sequencing, ChIP sequencing, and RNA-seq data have been deposited to NCBI GEO and are publicly available as of the date of publication. Accession numbers are listed in the key resources table. Additional female NPC clonal line RNA-seq data were taken from another study, and the GEO code can be found in the key resources table.^40^ This paper does not report original code Other data, materials, and analysis scripts are available upon request.

## Highlights

- Identification of 287 aRME genes in neural progenitor cells
- Genetic variation skews aRME of *Pvt1* through differential TFAP2a binding
- Epigenomic profiling reveals a bivalent promoter at aRME genes in development
- *Pvt1* monoallelic expression regulates the *Pvt1-Myc* loci to modulate cell proliferation
