# Supplementary material for: Genetic and chromatin regulation of Pvt1 monoallelic expression
Source: Cell Rep. Author manuscript; Available in PMC 2026 Jan 25. (PMC12832107; doi:10.1016/j.celrep.2025.116554)
Supplement: 1 [file NIHMS2125830-supplement-1.pdf]

Cell Reports, Volume *44*

Supplemental information

Genetic and chromatin regulation of *Pvt1* monoallelic expression

Christy Luong, Mason Chen, Julia A. Belk, Katerina Kraft, Anne-Valerie Gendrel, Edith Heard, Joanna Wysocka, and Howard Y. Chang

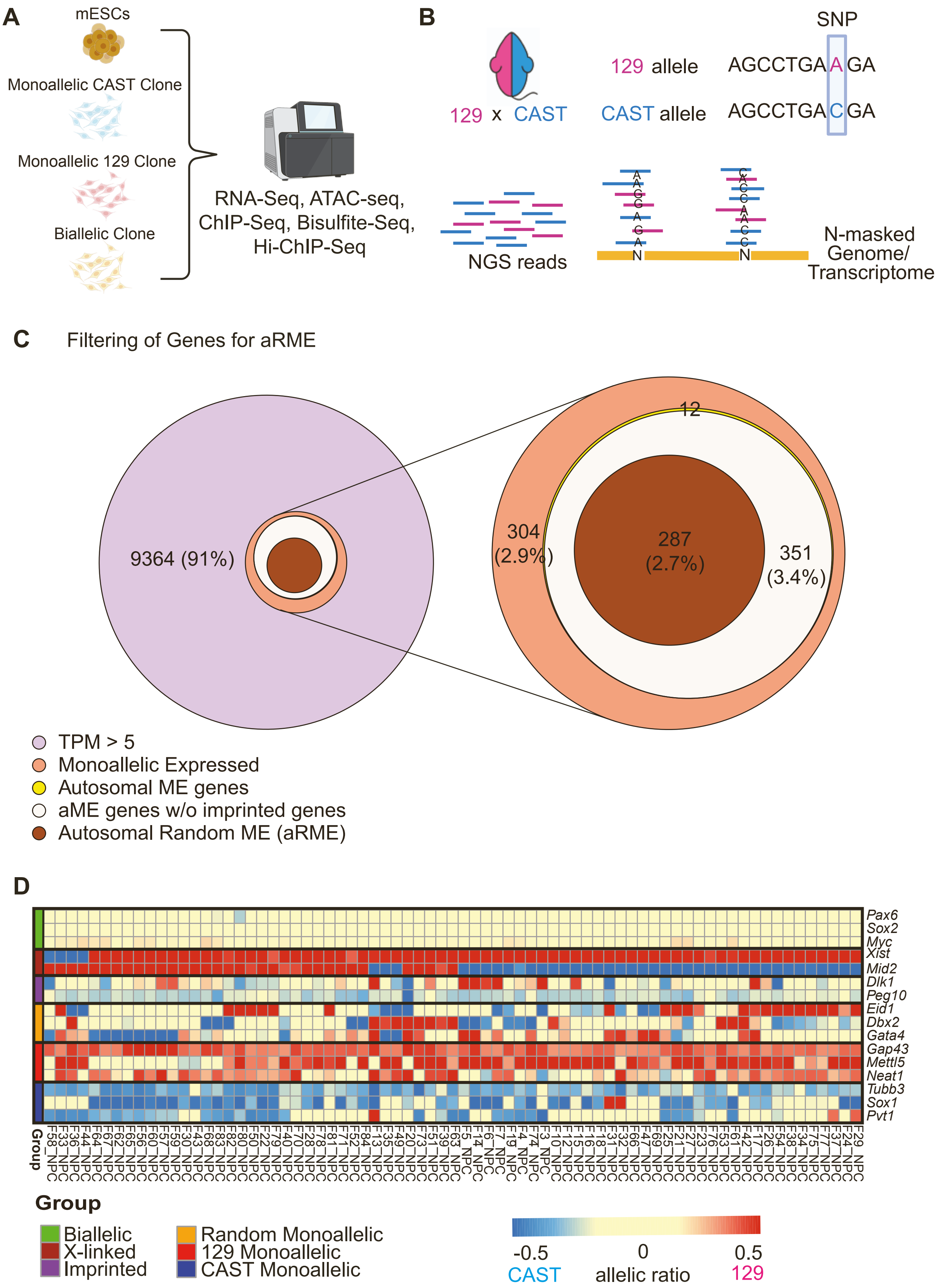
A
mESCs
Monoallelic CAST Clone
Monoallelic 129 Clone
Biallelic Clone
RNA-Seq, ATAC-seq,
ChIP-Seq, Bisulfite-Seq,
Hi-ChIP-Seq
B
129 x CAST
129 allele
CAST allele
SNP
AGCCTGA A GA
AGCCTGA C GA
NGS reads
N-masked
Genome/
Transcriptome
C
Filtering of Genes for aRME
9364 (91%)
12
304
(2.9%)
287
(2.7%)
351
(3.4%)
TPM > 5
Monoallelic Expressed
Autosomal ME genes
aME genes w/o imprinted genes
Autosomal Random ME (aRME)
D
Pax6
Sox2
Myc
Xist
Mid2
Dlk1
Peg10
Eid1
Dbx2
Gata4
Gap43
Mettl5
Neat1
Tubb3
Sox1
Pvt1
Group
Group
Biallelic
X-linked
Imprinted
Random Monoallelic
129 Monoallelic
CAST Monoallelic
-0.5
0
0.5
CAST
allelic ratio
129

Figure S1: Overview of monoallelic expressing genes

A) Experimental schematic of NGS experiments. mESCs and NPC clonal lines were expanded and used for down downstream library preps followed by next generation sequencing (NGS).

B) Analysis schematic for NGS experiments. Reads were mapped to a custom N-masked genome or transcriptome. Then aligned reads were split into genome 1 (129) or genome 2 (CAST) based on informative SNPs. If the reads did not contain an informative SNP, it was not used for allele-specific analysis.

C) A Venn diagram demonstrating the filtering performed to obtain genes that are known as aRME. In purple are all genes that are expressed in NPCs with average TPMs greater than 5 across all NPC clonal lines. On the right is a zoom in on the other four categories. Genes that are monoallelically expressed with AR cutoff of 0.3/-0.3 and has monoallelic expression (ME) of the gene in at least 40% of all clones (ME cutoff of 0.4) (orange). Autosomal ME genes with X and Y-linked genes removed (yellow). Autosomal ME genes with known imprinted genes filtered out (cream). Autosomal ME genes with at least one clonal line that is monoallelic CAST and one that is monoallelic 129. These genes are called autosomal random monoallelic expressing genes (aRME) (brown).

D) Heatmap of the AR of different genes across female NPC clonal lines. There are 6 groups of genes: biallelic (green), X-linked (brown), imprinted (purple), random monoallelic (RME) genes (orange), 129-biased monoallelic genes (red), and CAST-biased monoallelic genes (blue). The heatmap is colored from CAST-biased (blue) to biallelic (yellow) to 129-biased (red). On the X-axis is the name of each female clonal line (n=74).

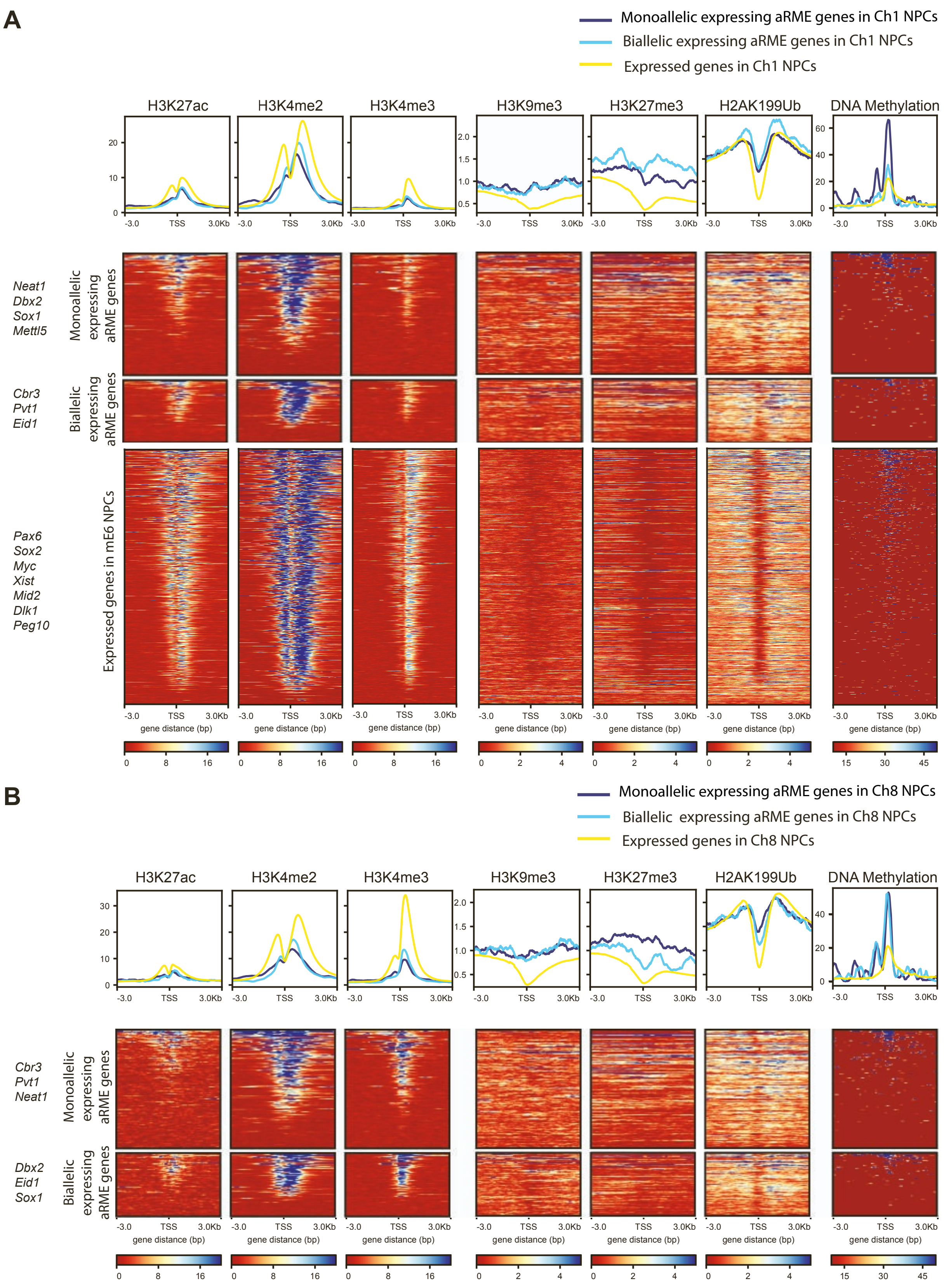
A
Monoallelic expressing aRME genes in Ch1 NPCs
Biallelic expressing aRME genes in Ch1 NPCs
Expressed genes in Ch1 NPCs
H3K27ac
H3K4me2
H3K4me3
H3K9me3
H3K27me3
H2AK199Ub
DNA Methylation
-3.0
TSS
3.0Kb
Neat1
Dbx2
Sox1
Mettl5
Monoallelic expressing aRME genes
Cbr3
Pvt1
Eid1
Biallelic expressing aRME genes
Pax6
Sox2
Myc
Xist
Mid2
Dlk1
Peg10
Expressed genes in mE6 NPCs
gene distance (bp)
B
Monoallelic expressing aRME genes in Ch8 NPCs
Biallelic expressing aRME genes in Ch8 NPCs
Expressed genes in Ch8 NPCs
Cbr3
Pvt1
Neat1
Monoallelic expressing aRME genes
Dbx2
Eid1
Sox1
Biallelic expressing aRME genes

Figure S2: Epigenomic profile of monoallelic expressing genes in other clonal NPC lines

A) Combined heatmap of chromatin immunoprecipitations (ChIPs) of epigenetic markers and bisulfite sequencing in the Ch1 NPC clonal line with the reference point centered around the transcriptional start site (TSS). There is H3K27ac, H3K4me3, H3K4me3, H3K9me3, H3K27me3, and H2AK199ub ChIPs along with DNA methylation from enriched bisulfite sequencing. At the top is summary plot of ChIP signals from the three groups of genes: monoallelic expressing aRME genes in Ch1 NPCs, biallelic expressing aRME in Ch1 NPCs, and all non aRME genes expressed in NPCs. Below the plot are the corresponding heatmaps created based on the gene grouping. On the left of the heatmap are some genes that can be found in each group. At the bottom is the scale in RPGC for the ChIPs and CPM for the DNA methylation.

B) Combined heatmap of chromatin immunoprecipitations (ChIPs) of epigenetic markers and bisulfite sequencing in the Ch8 NPC clonal line with the reference point centered around the transcriptional start site (TSS). There is H3K27ac, H3K4me3, H3K4me3, H3K9me3, H3K27me3, and H2AK199ub ChIPs along with DNA methylation from enriched bisulfite sequencing. At the top is summary plot of ChIP signals from the three groups of genes: monoallelic expressing aRME genes in Ch8 NPCs, biallelic expressing aRME in Ch8 NPCs, and all non aRME genes expressed in NPCs. Below the plot are the corresponding heatmaps created based on the two gene groupings. On the left of the heatmap are some genes that can be found in each group. At the bottom is the scale in RPGC for the ChIPs and CPM for the DNA methylation.

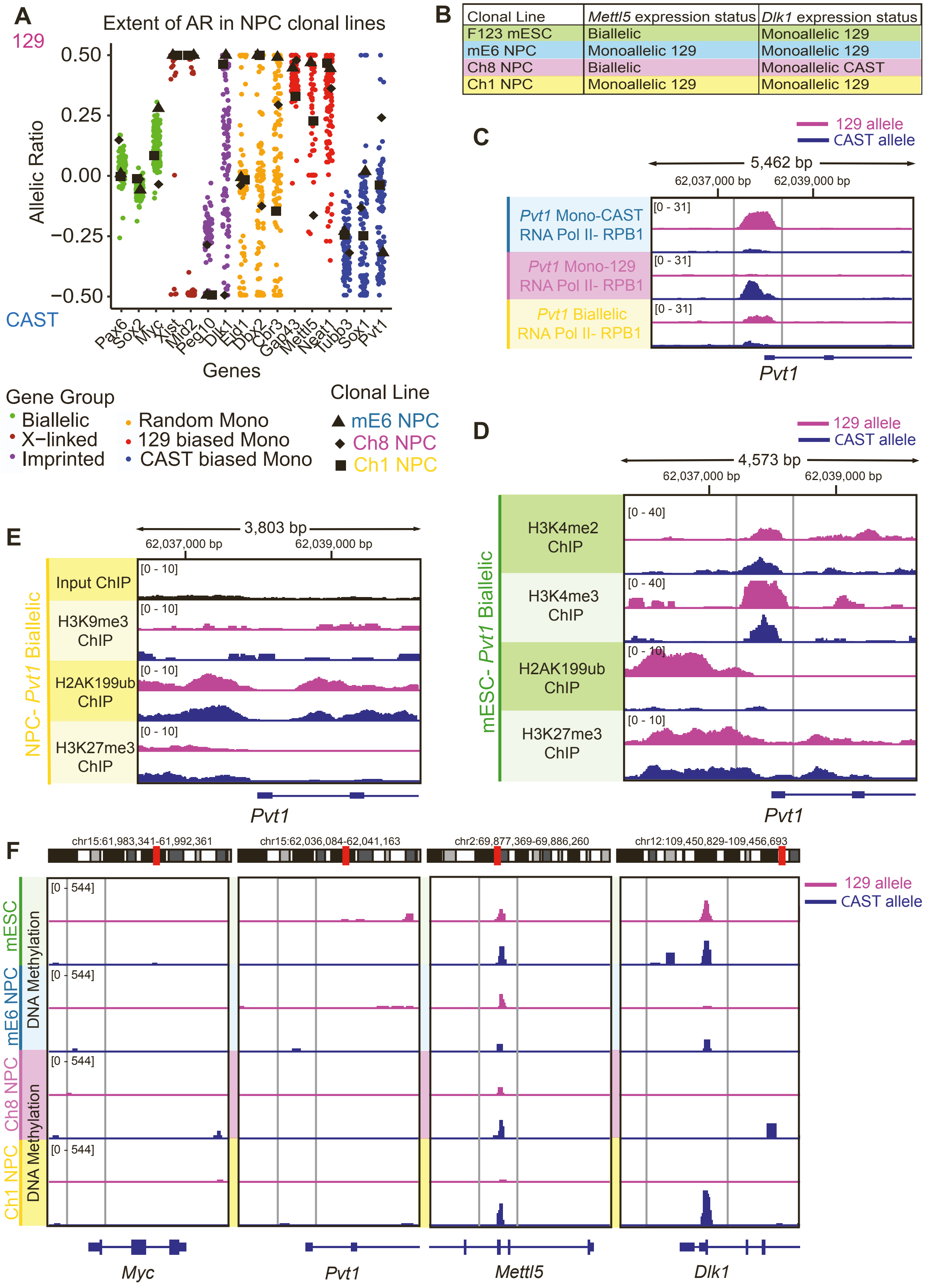

Clonal Line	*Mettl5* expression status	*Dlk1* expression status
F123 mESC	Biallelic	Monoallelic 129
mE6 NPC	Monoallelic 129	Monoallelic 129
Ch8 NPC	Biallelic	Monoallelic CAST
Ch1 NPC	Monoallelic 129	Monoallelic 129

Figure S3: Further characterization of the chromatin landscape at the *Pvt1* locus

A) Allelic Ratio (AR) of *Pvt1* in F_1-23 Hybrid NPC clonal lines compared to AR of biallelic genes (green), X-linked genes (brown), aRME genes (orange), 129-biased genes (red), and other CAST-biased genes (blue). Each point represents a clonal line (n=120). NPC clonal lines used for other experiments are highlighted with different shapes.

B) Summary of F_1 hybrid clonal lines used in this paper, and their respective *Mettl5* and *Dlk1* allelic expression status based off of an AR cutoff of 0.2.

C) Allele-specific RNA Pol II subunit RPB1ChIP-seq from 3 different F_1-23 clonal lines: NPC clonal line with *Pvt1* monoallelic CAST expression (blue), NPC clonal line with *Pvt1* monoallelic 129 expression (pink), and NPC clonal line with *Pvt1* biallelic expression (yellow). Signals around *Pvt1*. Peak signals of interest are highlighted with the grey box. Reads assigned to 129-allele in pink and CAST-allele in blue. Data range in RPGC.

D) Allele-specific H3K4me2, H3K4me3, H2AK119Ub, and H3K27me3 ChIP-seq from F_1-23 mESCs with *Pvt1* biallelic expression (green). Signals around *Pvt1*. Peak signals of interest are highlighted with the grey box. Reads assigned to 129-allele in pink and CAST-allele in blue. Data range for in RPGC.

E) Allele-specific input, H3K9me3, H2AK119Ub, and H3K27me3 ChIP-seq from Ch1 NPCs, clonal line with *Pvt1* biallelic expression (yellow). Signals around *Pvt1*. Reads assigned to 129-allele in pink and CAST-allele in blue. Data range in RPGC.

F) Allele-specific bisulfite DNA methylation signals from Illumina TruSeq Methyl Capture Epic Library prep. Four different samples: mESCs (green), NPC clonal line with *Pvt1* expressed from CAST-allele (blue), NPC clonal line with *Pvt1* expressed from 129-allele (pink), and NPC clonal line with *Pvt1* expressed from both alleles (yellow). Signals around the *Myc, Pvt1, Mettl5,* and *Dlk1*. Peak signals of interest are highlighted with the grey box. Reads assigned to 129-allele in pink and CAST-allele in blue. Data range in CPM.

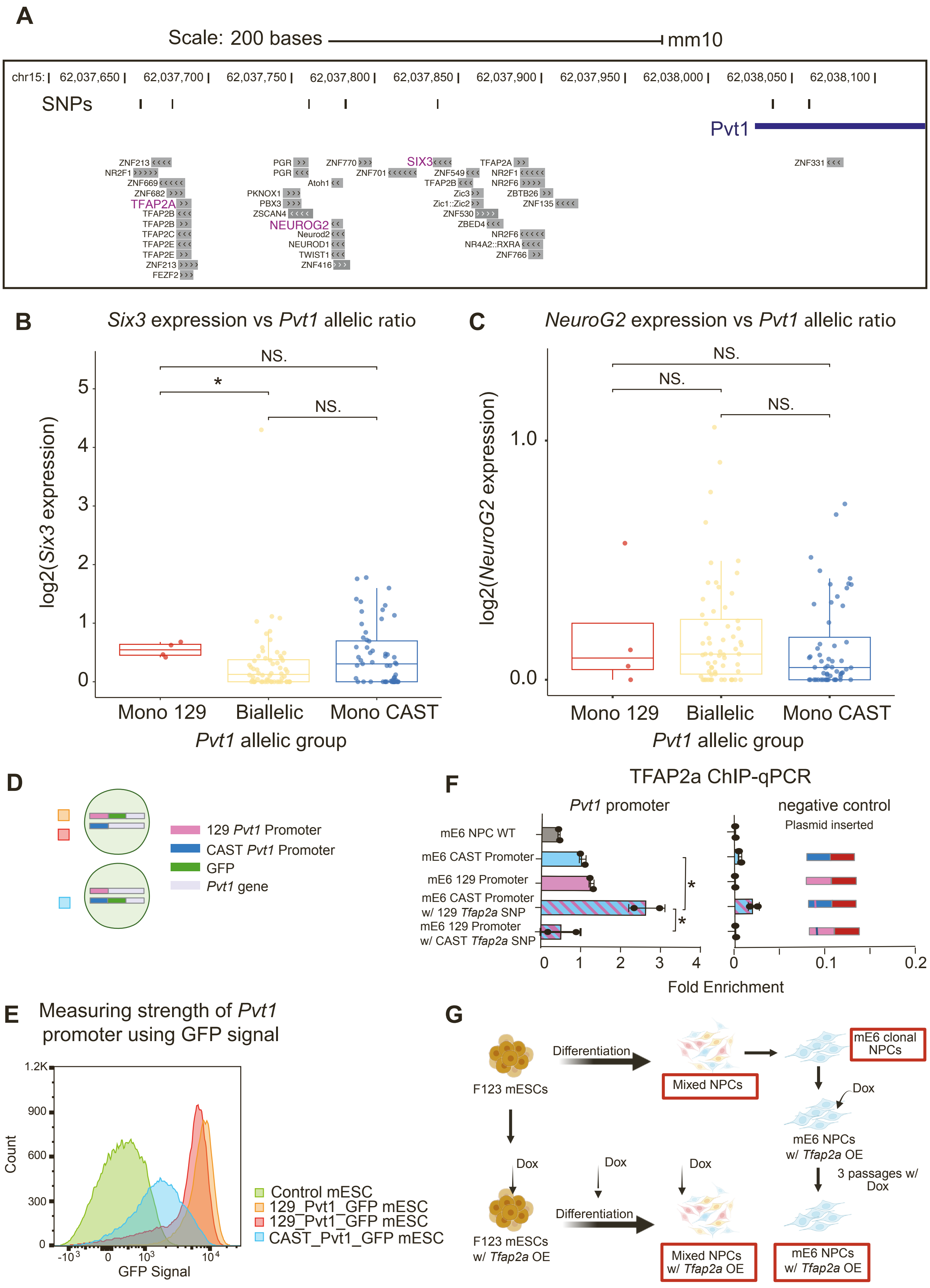
A
Scale: 200 bases
mm10
chr15: 62,037,650 62,037,700 62,037,750 62,037,800 62,037,850 62,037,900 62,037,950 62,038,000 62,038,050 62,038,100
SNPs
Pvt1
ZNF213 NR2F1 ZNF669 ZNF682 TFAP2A TFAP2B TFAP2B TFAP2C TFAP2E TFAP2E ZNF213 FEZF2
PGR PGR Atoh1 PKNOX1 PBX3 ZSCAN4 NEUROG2 Neurod2 NEUROD1 TWIST1 ZNF416
ZNF770 ZNF701 SIX3 ZNF549 TFAP2B Zic3 Zic1::Zic2 ZNF530 ZBED4
TFAP2A NR2F1 NR2F6 ZBTB26 ZNF135 NR2F6 NR4A2::RXRA ZNF766
ZNF331
B
Six3 expression vs Pvt1 allelic ratio
log2(Six3 expression)
NS.
*
NS.
Mono 129
Biallelic
Mono CAST
Pvt1 allelic group
C
NeuroG2 expression vs Pvt1 allelic ratio
log2(NeuroG2 expression)
NS.
NS.
NS.
Mono 129
Biallelic
Mono CAST
Pvt1 allelic group
D
129 Pvt1 Promoter
CAST Pvt1 Promoter
GFP
Pvt1 gene
E
Measuring strength of Pvt1 promoter using GFP signal
Count
GFP Signal
Control mESC
129_Pvt1_GFP mESC
129_Pvt1_GFP mESC
CAST_Pvt1_GFP mESC
F
TFAP2a ChIP-qPCR
Pvt1 promoter
negative control
Plasmid inserted
mE6 NPC WT
mE6 CAST Promoter
mE6 129 Promoter
mE6 CAST Promoter w/ 129 Tfap2a SNP
mE6 129 Promoter w/ CAST Tfap2a SNP
Fold Enrichment
G
F123 mESCs
Differentiation
Mixed NPCs
mE6 clonal NPCs
Dox
mE6 NPCs w/ Tfap2a OE
3 passages w/ Dox
F123 mESCs w/ Tfap2a OE
Mixed NPCs w/ Tfap2a OE
mE6 NPCs w/ Tfap2a OE

Figure S4: 129 Pvt1 promoter is stronger than Cast promoter in mESCs

A) Genomic track centered around *Pvt1* promoter with potential transcriptional factor (TF) binding sites from JASPAR. On the top row are the SNPs found between 129-allele and CAST-allele. Some TFs that are expressed and found near or on a SNP are colored in purple.

B) *Six3* expression from F_1-23 hybrid NPC clonal lines. Clonal lines are grouped based on *Pvt1* allelic expression status: Mono CAST - AR ≤ -0.2 (n=56, blue), Biallelic- AR between -0.2 and 0.2 (n= 60, yellow), Mono 129- AR ≥ 0.2 (n=4, red). Significance calculated with t-test: “NS” for p-value > 0.05, and “*” for p-value ≤ 0.05.

C) *NeuroG2* expression from F_1-23 hybrid NPC clonal lines. Clonal lines are grouped based on *Pvt1* allelic expression status: Mono CAST - AR ≤ -0.2 (n=56, blue), Biallelic- AR between -0.2 and 0.2 (n=60, yellow), Mono 129- AR ≥ 0.2 (n=4, red). Significance calculated with t-test: “NS” for p-value > 0.05.

D) Experimental design for mESC clonal lines with *Pvt1* promoter endogenously fused to GFP. In two clonal lines, the 129 *Pvt1* Promoter is fused to GFP, and the CAST *Pvt1* promoter is untouched. In another clone, CAST *Pvt1* promoter is fused to GFP and 129 *Pvt1* Promoter is untouched.

E) A flow cytometry histogram of four different mESC clonal lines and their GFP signal. The green is a control mESC clonal line with no GFP. In orange and red are two different mESC clonal lines with 129 *Pvt1* promoter fused to GFP. In blue is an mESC clonal line with CAST *Pvt1* promoter fused to GFP as described in **Supp.Fig. 4D**.

F) Fold enrichment plot for TFAP2a ChIP-qPCR with five different samples: mE6 NPC and four mE6 NPC with transient transfection of a plasmid. All plasmids contain red fluorescent protein for normalization. The plasmids are CAST *Pvt1* promoter (blue), 129 *Pvt1* promoter (pink), CAST *Pvt1* promoter with altered TFAP2a SNP (G) to the 129 SNP (A) (blue base with pink strips), and 129 *Pvt1* promoter with altered TFAP2a SNP (A) to the CAST SNP (G) (pink base with blue strips). The left plot is a qPCR for the *Pvt1* promoter region, and the right plot is a qPCR for a negative control region (B2 SINE region). Significance calculated with t-test: “NS” for p-value > 0.05, and “*” for p-value ≤ 0.05.

G) Experimental schematic for **Fig. 5G.** F_1-23 mESCs were transduced with lentivirus packaged with a dox-inducible plasmid for *Tfap2a* overexpression. Doxycycline was then added to the mESCs throughout the differentiation process to mix NPCs, as previously described. mE6 NPCs were transduced with lentivirus packaged with a dox-inducible plasmid for *Tfap2a* overexpression. The NPCs were then passaged three times with the addition of doxycycline. Samples highlighted by the red box were collected for targeted RNA-seq.

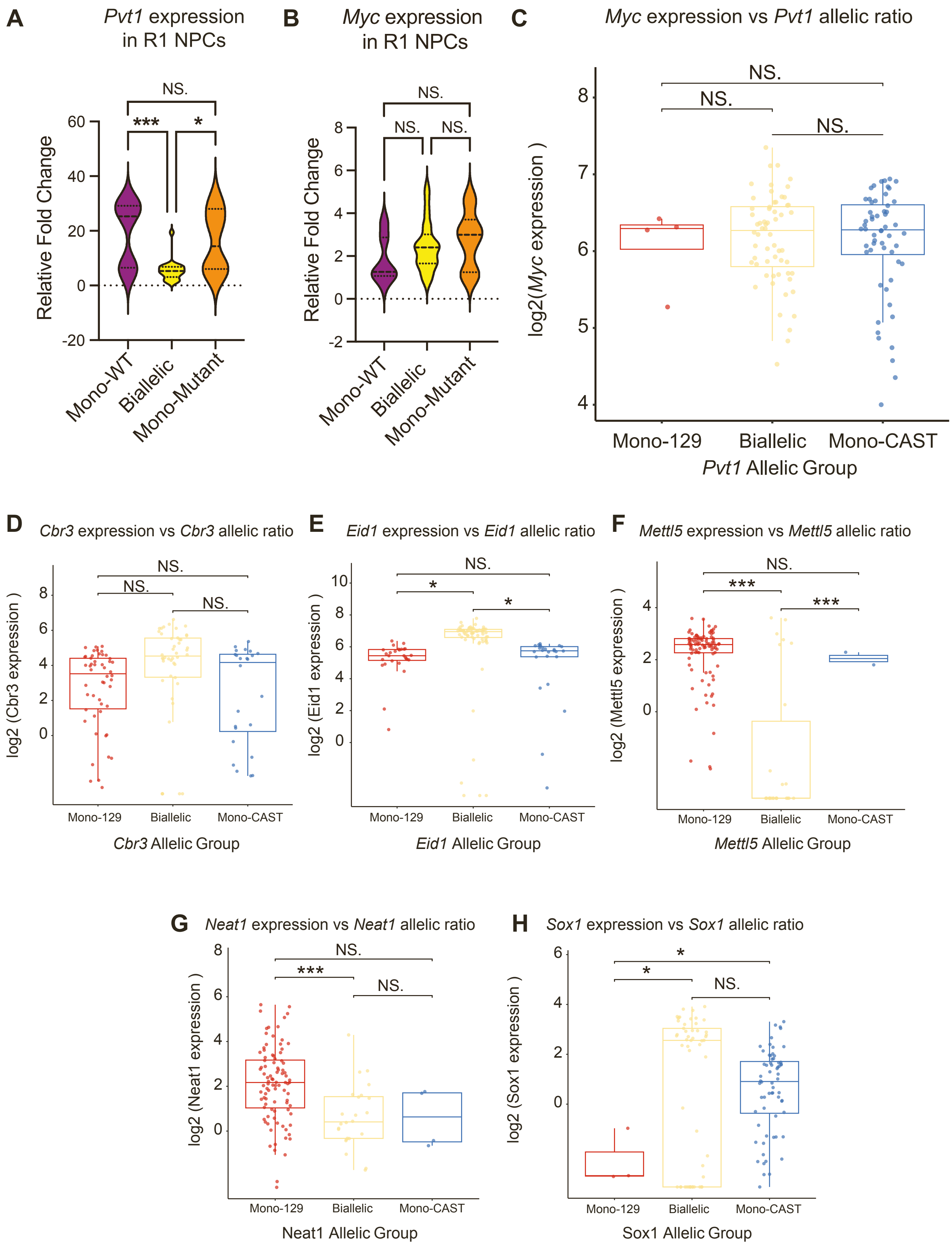
A
Pvt1 expression in R1 NPCs
NS.
***
*
Relative Fold Change
60
40
20
0
-20
Mono-WT
Biallelic
Mono-Mutant
B
Myc expression in R1 NPCs
NS.
NS.
NS.
Relative Fold Change
8
6
4
2
0
-2
Mono-WT
Biallelic
Mono-Mutant
C
Myc expression vs Pvt1 allelic ratio
NS.
NS.
NS.
log2(Myc expression)
8
7
6
5
4
Mono-129
Biallelic
Mono-CAST
Pvt1 Allelic Group
D
Cbr3 expression vs Cbr3 allelic ratio
NS.
NS.
NS.
log2 (Cbr3 expression)
8
6
4
2
0
Mono-129
Biallelic
Mono-CAST
Cbr3 Allelic Group
E
Eid1 expression vs Eid1 allelic ratio
NS.
*
*
log2 (Eid1 expression)
10
8
6
4
2
0
Mono-129
Biallelic
Mono-CAST
Eid1 Allelic Group
F
Mettl5 expression vs Mettl5 allelic ratio
NS.
***
***
log2 (Mettl5 expression)
4
2
0
Mono-129
Biallelic
Mono-CAST
Mettl5 Allelic Group
G
Neat1 expression vs Neat1 allelic ratio
NS.
***
NS.
log2 (Neat1 expression)
8
6
4
2
0
Mono-129
Biallelic
Mono-CAST
Neat1 Allelic Group
H
Sox1 expression vs Sox1 allelic ratio
*
*
NS.
log2 (Sox1 expression)
6
4
2
0
Mono-129
Biallelic
Mono-CAST
Sox1 Allelic Group

Figure S5: *Pvt1* and *Myc* expression in NPC clonal lines

A) *Pvt1* expression from RT-qPCR of R1-57 NPC clonal lines. Clonal lines with clones grouped based on *Pvt1* allelic expression status: Monoallelic for WT allele is AR ≥ 0.2 (n=12), Biallelic if AR is between 0.2 and -0.2 (n=29), and Monoallelic for mutant allele if AR ≤ 0.2 (n=6). Relative fold change compared to NPC clonal line with lowest *Pvt1* expression. Significance calculated with t- test: "NS" for p-value > 0.05, "*" for p-value ≤ 0.05, and "***" for p-value ≤ 0.001.

B) *Myc* expression from RT-qPCR of R1-57 NPC clonal lines. Clonal lines with clones grouped based on *Pvt1* allelic expression status: Monoallelic for WT allele is AR ≥ 0.2 (n=12), Biallelic if AR is between 0.2 and -0.2 (n=29), and Monoallelic for mutant allele if AR ≤ 0.2 (n=6). Relative fold change compared to NPC clonal line with lowest *Myc* expression. Significance calculated with t-test: "NS" for p-value > 0.05.

C) *Myc* expression in F_1-23 NPC clonal lines. Clonal lines are grouped based on *Pvt1* allelic expression status: Mono CAST- AR ≤-0.2 (n=56, blue), Biallelic- AR between -0.2 and 0.2 (n=60, yellow), Mono 129- AR ≥0.2 (n=4, red). Significance calculated with t-test: "NS" for p-value > 0.05.

D) *Cbr3* expression in F_1-23 NPC clonal lines. Clonal lines are grouped based on *Cbr3* allelic expression status: Mono CAST- AR ≤-0.2 (n=24, blue), Biallelic- AR between -0.2 and 0.2 (n=46, yellow), Mono 129- AR ≥0.2 (n=50, red). Significance calculated with t-test: "NS" for p-value > 0.05 and "*" for p-value ≤ 0.05.

E) *Eid1* expression in F_1-23 NPC clonal lines. Clonal lines are grouped based on *Eid1* allelic expression status: Mono CAST- AR ≤-0.2 (n=25, blue), Biallelic- AR between -0.2 and 0.2 (n=68, yellow), Mono 129- AR ≥0.2 (n=27, red). Significance calculated with t-test: "NS" for p-value > 0.05 and "**" for p-value ≤ 0.01.

F) *Mettl5* expression in F_1-23 NPC clonal lines. Clonal lines are grouped based on *Mettl5* allelic expression status: Mono CAST- AR ≤-0.2 (n=2, blue), Biallelic- AR between -0.2 and 0.2 (n=26, yellow), Mono 129- AR ≥0.2 (n=92, red). Significance calculated with t-test: "NS" for p-value > 0.05, "**" for p-value ≤ 0.01, and "***" for p-value ≤ 0.001.

G) *Neat1* expression in F_1-23 NPC clonal lines. Clonal lines are grouped based on *Neat1* allelic expression status: Mono CAST- AR ≤-0.2 (n=4, blue), Biallelic- AR between -0.2 and 0.2 (n=24, yellow), Mono 129- AR ≥0.2 (n=92, red). Significance calculated with t-test: "NS" for p-value > 0.05 and "***" for p-value ≤ 0.001.

H) *Sox1* expression in F_1-23 NPC clonal lines. Clonal lines are grouped based on *Sox1* allelic expression status: Mono CAST- AR ≤-0.2 (n=64, blue), Biallelic- AR between -0.2 and 0.2 (n=53, yellow), Mono 129- AR ≥0.2 (n=3, red). Significance calculated with t-test: "*" for p-value ≤ 0.05 and "***" for p-value ≤ 0.001.
